# Supplementary material for: The MinDE system is a generic spatial cue for membrane protein distribution in vitro
Source: Nat Commun. 2018 Sep 26;9:3942. doi: 10.1038/s41467-018-06310-1 (PMC6158289; doi:10.1038/s41467-018-06310-1)
Supplement: Supplementary file 1 — Supplementary Information [file 41467_2018_6310_MOESM1_ESM.pdf]

Supplementary Information

The MinDE system is a generic spatial cue for membrane protein distribution in vitro

Beatrice Ramm[1], Philipp Glock[1], Jonas Mücksch[1], Philipp Blumhardt[1], Daniela A. García-Soriano[1], Michael Heymann[1], Petra Schwille[1,*]

[1] Max Planck Institute of Biochemistry, Am Klopferspitz 18, D-82152 Martinsried, Germany.

Correspondence and requests for materials should be addressed to P.S. (email: schwille@biochem.mpg.de)

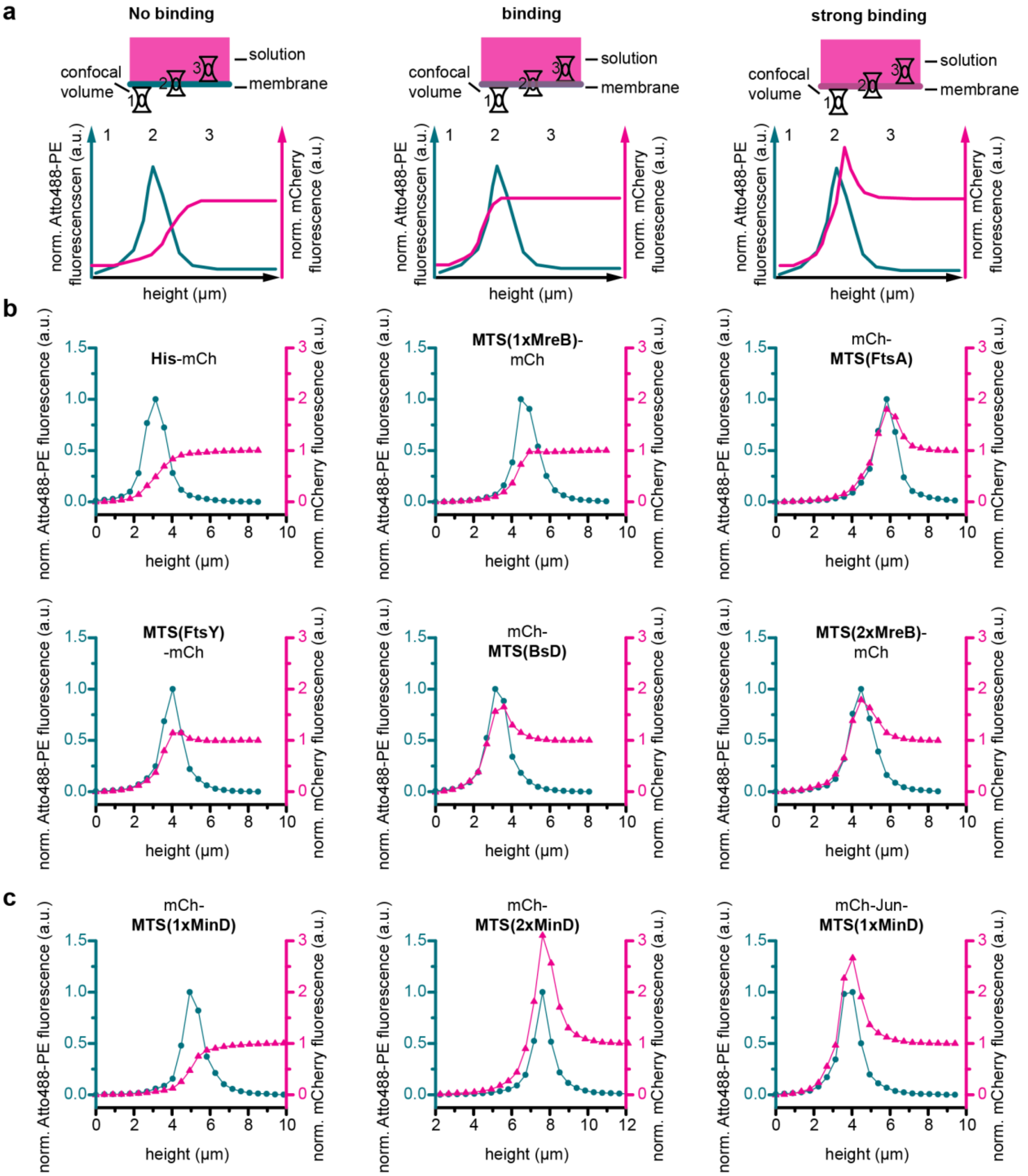

Supplementary Figure 1: Model peripheral membrane proteins, mCh-MTS constructs, bind to the membrane. a) Schematic explaining the experimental setup. Z-stacks of an assay chamber with a fluorescently labeled supported lipid bilayer (cyan) and the corresponding mCh-MTS constructs (magenta) are acquired (supported lipid bilayer (SLB): 70 mol % DOPC, 30 mol % DOPG, 0.05 mol % Atto655-PE, 1 µM mCh-MTS). If no binding occurs, the fluorescence intensity signal of the mCh-MTS construct reaches its maximum spatially above the signal from the labeled SLB, when the confocal volume fully entered the solution. If weak binding occurs the maximum of the fluorescence intensity signal of the mCh-MTS coincides with the maximum of the SLB signal, as some of the protein is located on the membrane. If strong binding occurs and the mCh-MTS construct accumulates on the membrane, the signal of the mCh-MTS construct is higher on the membrane than in solution. b) Representative z-stacks of mCh-MTS constructs confirming binding of all mCh-MTS constructs except His-mCh. c) Representative z-stacks of mCh-MTS constructs harboring the *E. coli* MinD membrane targeting sequence (MTS). No binding can be detected for mCh-MTS(1xMinD), but strong binding for mCh-MTS(2xMinD) and mCh-Jun-MTS(1xMinD). mCh-MTS fluorescence (magenta triangles) is normalized to the first two values below the membrane and the last two values in solution. Atto-655-PE fluorescence (cyan spheres) is normalized to the minimal and maximal values.

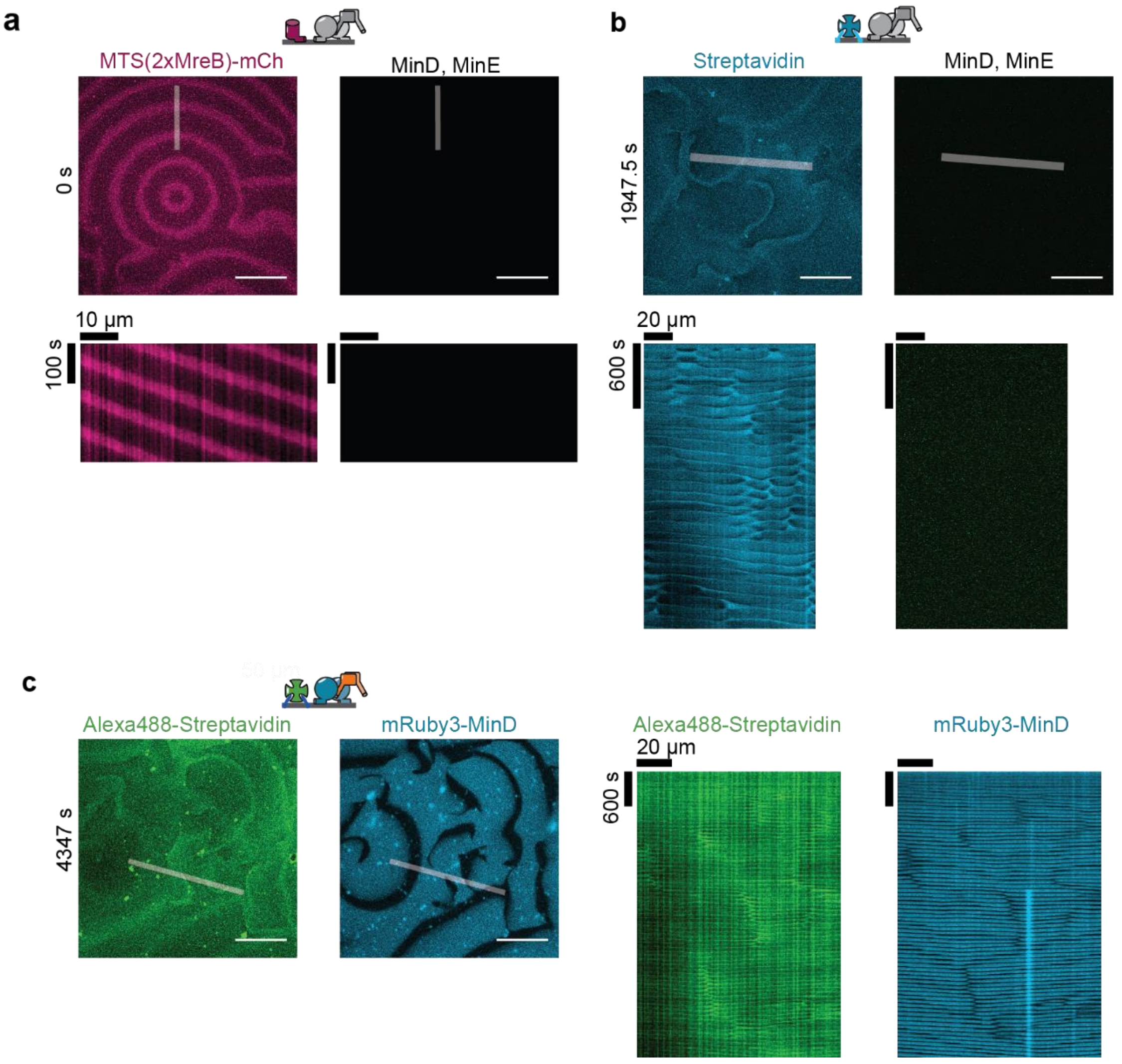

Supplementary Figure 2: Spatiotemporal regulation of MinDE is not a photoartifact. a) Positioning of MTS(2xMreB)-mCh by MinDE in the absence of labeled MinD/MinE (1 µM MinD, 1 µM MinE, 1 µM MTS(2xMreB)-mCh, 2.5 mM ATP). b) Regulation of Alexa647-streptavidin anchored to biotinylated lipids by MinDE in the absence of labeled MinD/MinE (1 µM MinD, 1 µM MinE, Alexa647-streptavidin) c) Positioning of Alexa488-streptavidin by MinDE (1 µM MinD (30 % mRuby3-MinD), 1 µM MinE, Alexa488-streptavidin). Experiments were performed independently two (b and c) or three (a) times under identical or similar conditions.

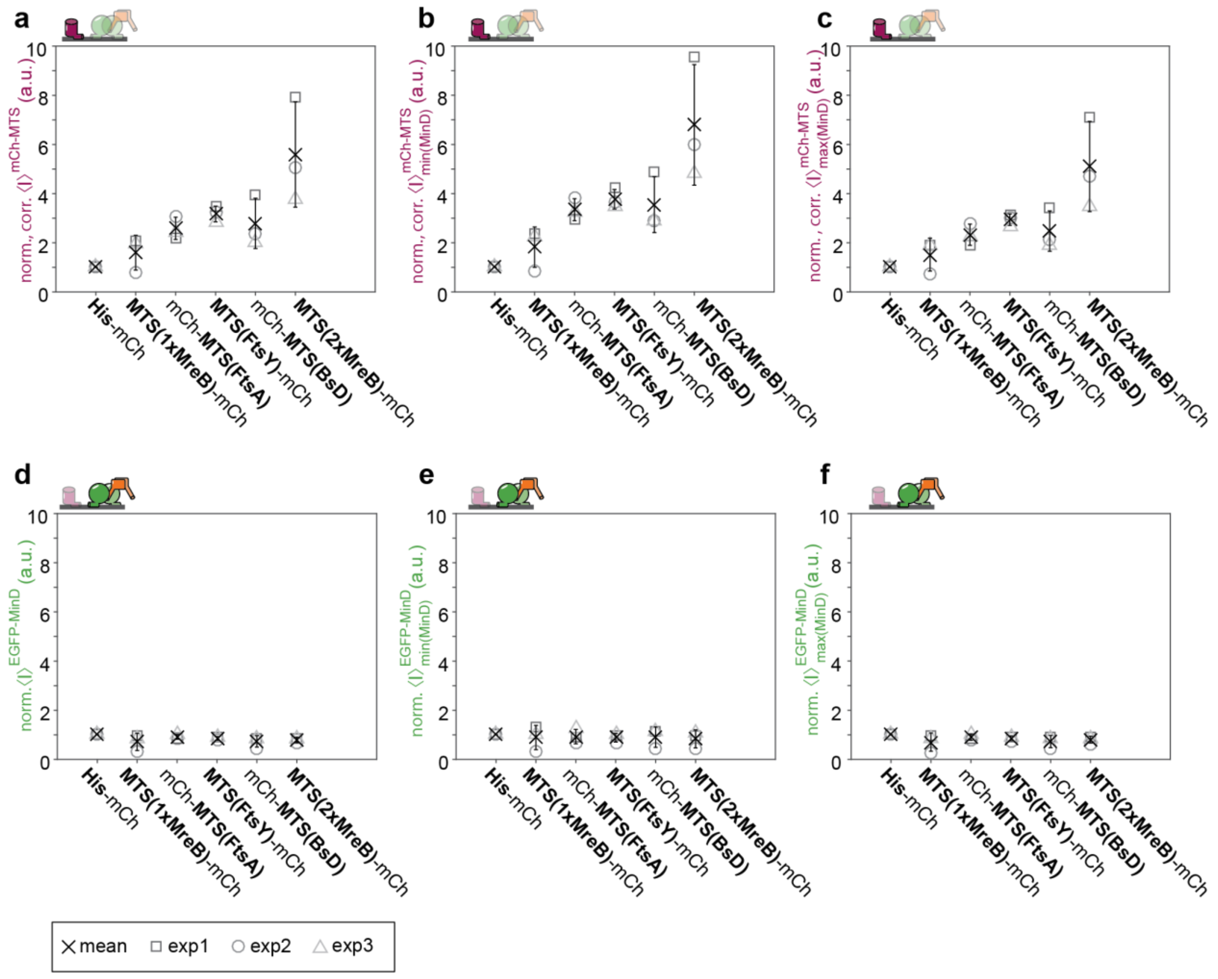

Supplementary Figure 3: mCh-MTS constructs have different membrane affinities, but do not influence the membrane density of EGFP-MinD. a)-c) mCh-MTS intensity on the membrane, i.e. membrane affinity, increases from MTS(1xMreB)-mCh to MTS(2xMreB)-mCh. a) Average mCh-MTS intensity of the full image normalized to His-mCh and corrected for the fluorescent protein fraction ($\langle I \rangle^{\mathrm{mCh-MTS}}$). b) Average mCh-MTS intensity in the MinDE wave minimum normalized to His-mCh and corrected for the fluorescent protein fraction ($\langle \boldsymbol{I} \rangle^{\mathrm{mCh\text{-}MTS}}_{\mathbf{min(D)}}$). c) Average mCh-MTS intensity in the MinDE wave maximum normalized to His-mCh and corrected for the fluorescent protein fraction ($\langle \boldsymbol{I} \rangle^{\mathrm{mCh\text{-}MTS}}_{\mathbf{max(D)}}$) (as shown in Fig. 2d). d)-f) EGFP-MinD intensity, i.e. density, on the membrane is not influenced by the addition of mCh-MTS constructs. d) Average EGFP-MinD intensity of the full image normalized to the fluorescence in the presence of His-mCh ($\langle I \rangle^{\mathrm{EGFP-MinD}}$). e) Average EGFP-MinD intensity in the MinDE wave minimum normalized to the fluorescence in the presence of His-mCh ($\langle \boldsymbol{I} \rangle^{\mathrm{EGFP\text{-}MinD}}_{\mathbf{min(D)}}$). f) Average EGFP-MinD intensity in the MinDE wave maximum normalized to the fluorescence in the presence of His-mCh ($\langle \boldsymbol{I} \rangle^{\mathrm{EGFP\text{-}MinD}}_{\mathbf{max(D)}}$). Each data point (squares, spheres, triangles) (exp1-3) was generated from at least one tile scan (7 by 7) in one chamber. Cross and error bars represent the mean value and standard deviation of the three independent experiments with in total N images ($N_{\textbf{His-mCh}}$ = 343, $N_{\mathrm{MTS(1xMreB)\text{-}mCh}}$ = 294, $N_{\mathrm{mCh\text{-}MTS(FtsA)}}$ = 490, $N_{\mathrm{MTS(FtsY)\text{-}mCh}}$ = 392, $N_{\mathrm{mCh\text{-}MTS(BsD)}}$ = 390, $N_{\mathrm{MTS(2xMreB)\text{-}mCh}}$ = 265).

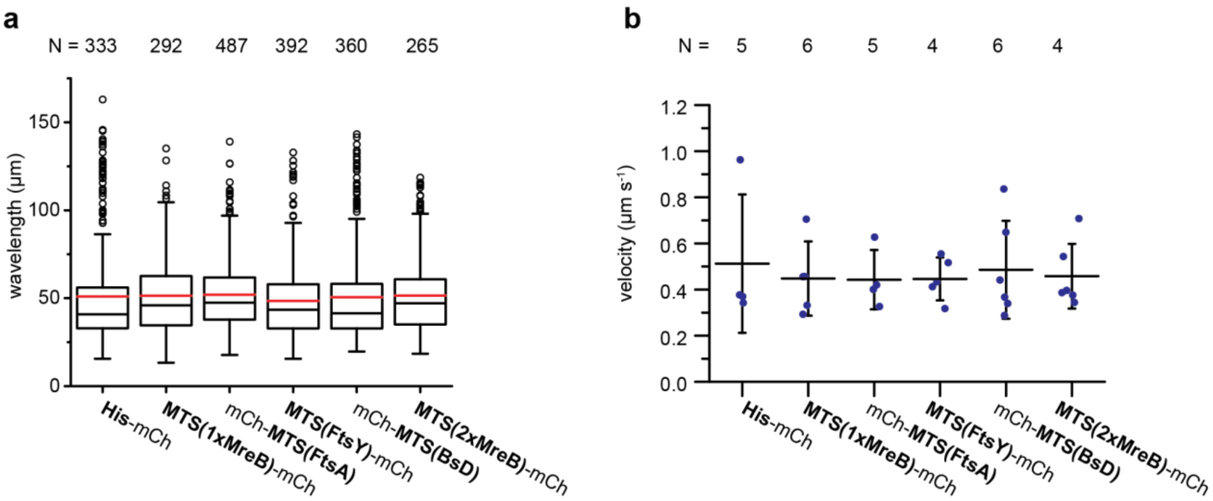

Supplementary Figure 4: The presence of model peripheral membrane proteins, mCh-MTS, does not change the wavelength or velocity of MinDE waves. a) Box plot of wavelength of the MinDE waves in the presence of the different mCh-MTS constructs. Box limits are quartiles 1 and 3 and whiskers are 1.5 x IQR, median is shown as a black line, mean is shown as a red line, points are outliers. (Data from three independent experiments shown in Figure 2b, with in total N measurements ($N_{\textbf{His-mCh}}$ = 333, $N_{\text{MTS(1xMreB)-mCh}}$ = 292s, $N_{\text{mCh-MTS(FtsA)}}$ = 487, $N_{\text{MTS(FtsY)-mCh}}$ = 392, $N_{\text{mCh-MTS(BsD)}}$ = 360, $N_{\text{MTS(2xMreB)-mCh}}$ = 265). b) Velocity of MinDE waves in the presence of different mCh-MTS constructs. Line and error bars represent mean and standard deviation from three independent experiments shown in Figure 2 with measurements from N different time-series ($N_{\text{His-mCh}}$ = 5, $N_{\text{MTS(1xMreB)-mCh}}$ = 6, $N_{\text{mCh-MTS(FtsA)}}$ = 5, $N_{\text{MTS(FtsY)-mCh}}$ = 4, $N_{\text{mCh-MTS(BsD)}}$ = 6, $N_{\text{MTS(2xMreB)-mCh}}$ = 4).

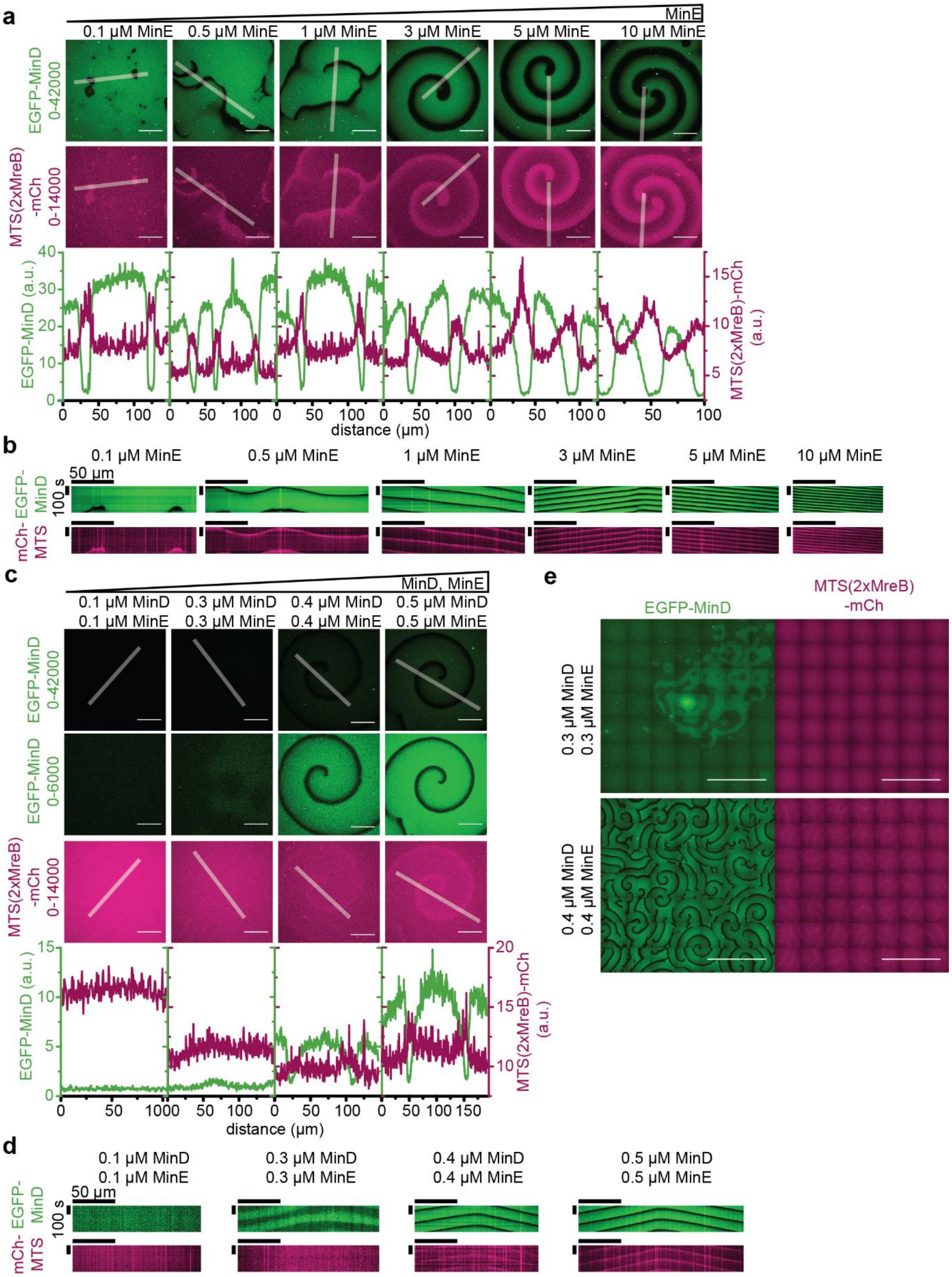

Supplementary Figure 5: MTS(2xMreB)-mCh is spatiotemporally regulated over a wide concentration range of MinD and MinE. a) MTS(2xMreB)-mCh is spatiotemporally regulated at different MinD/MinE ratios (1 µM MinD (30 % EGFP-MinD), 0.1 - 10 µM MinE, 1 µM MTS(2xMreB)-mCh). Representative images of MinDE self-organization (upper panel, green) in the presence of MTS(2xMreB)-mCh (middle panel, magenta). Fluorescence intensity line plots along the selection shown in the corresponding images (not necessarily full selection) (lowest panel). All images in one row were acquired and displayed using the same instrumental settings. Scale bars: 50 µm. b) Kymograph along the line selections shown in a). Scale bars: 100 s and 50 µm. c) MTS(2xMreB)-mCh is spatiotemporally regulated at all MinDE concentrations where MinDE are reliably self-organizing on the

membrane (>0.4 µM MinD and MinE). Representative images of MinDE self-organization in two different brightness settings (two upper panels) in the presence of MTS(2xMreB)-mCh (lower panel). Fluorescence intensity line plots along the selection shown in the corresponding images (not necessarily full selection) (lowest panel). All images in one row were acquired and displayed using the same instrumental settings (0.1 - 0.5 µM MinD (30 % EGFP-MinD), 0.1 - 0.5 µM MinE, 1 µM MTS(2xMreB)-mCh). Scale bars: 50 µm. d) Kymograph along the line selections shown in c) Scale bars: 100 s and 50 µm. e) MinDE only reliably self-organize at concentrations equal or higher than 0.4 µM MinDE. Representative tile scan of chambers containing 0.3 µM and 0.4 µM MinDE and 1 µM MTS(2xMreB)-mCh. At 0.3. µM MinDE patterns only form transiently in certain areas of the chamber, whereas at 0.4 µM MinDE patterns form reliably and also spatiotemporally regulate MTS(2xMreB)-mCh. Brightness/contrast settings are not comparable between images. Scale bars: 500 µm. All experiments were performed independently two times under similar or identical conditions.

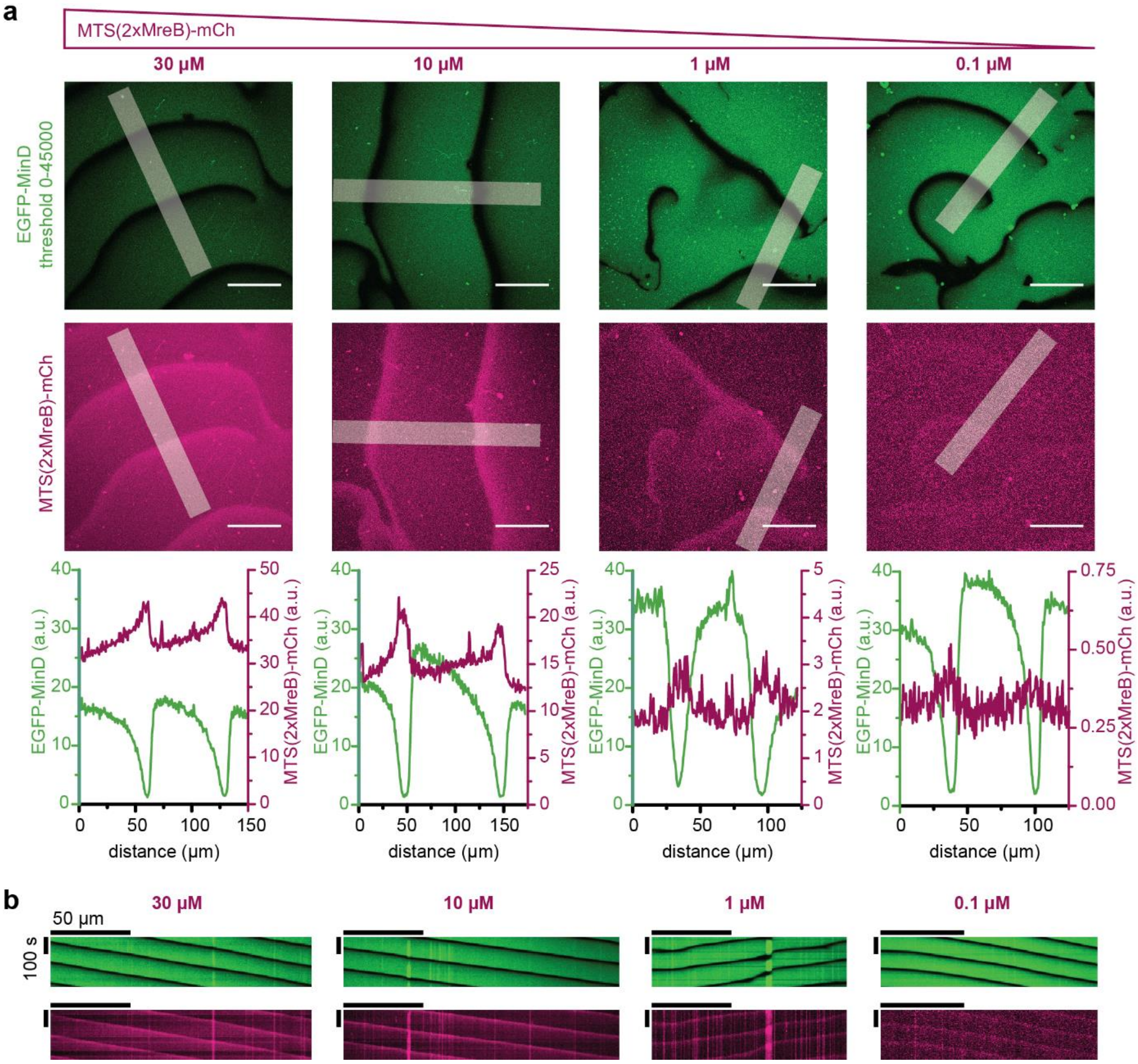

Supplementary Figure 6: MTS(2xMreB)-mCh is spatiotemporally regulated by MinDE over a wide MTS(2xMreB)-mCh/MinDE ratio. a) Representative images of the MinDE wave (upper panel, green) and the anticorrelated MTS(2xMreB)-mCh waves (lower panel, magenta) on the membrane (30 µM, 10 µM, 1 µM or 0.1 µM MTS(2xMreB)-mCh, 1 µM MinD (30 % EGFP-MinD), 1 µM MinE). All images in one row were acquired using the same imaging settings, MTS(2xMreB)-mCh brightness/constrast settings were optimized for each image and are not comparable. Fluorescence intensity line plots along the selection shown in the corresponding images (not necessarily full selection) (lowest panel). Scale bars: 50 µm. b) Kymograph along the line selections shown in a), but with line width 10 instead of the displayed line width 50. Scale bars: 100 s and 50 µm. Experiments were performed independently three times under identical conditions.

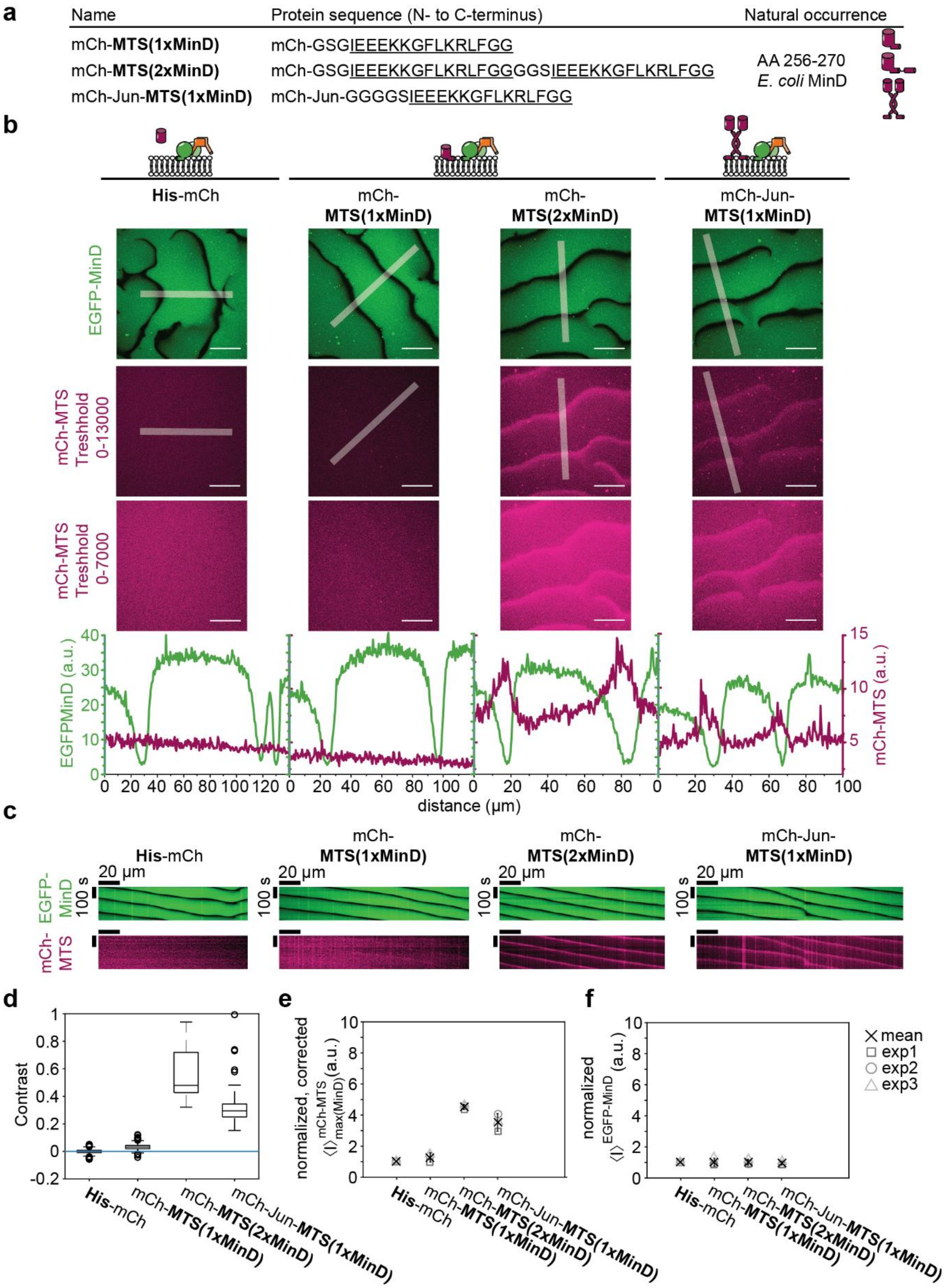

Supplementary Figure 7: Model membrane proteins containing two copies of the *E. coli* MinD amphipathic helix are spatiotemporally regulated by MinDE. a) Overview over the mCherry fusions to the *E. coli* MinD MTS. b) Representative images of the MinDE wave (upper panel, green) and the mCh-MTS constructs in two different brightness settings (middle and lower panels, magenta) on the membrane (1 µM mCh-MTS, 1 µM MinD (30 % EGFP-MinD), 1 µM MinE). All images in one row were acquired and displayed using the same instrumental settings. Fluorescence intensity line plots along the selection shown in the corresponding images (not necessarily full selection) show the difference in the extent of the spatial regulation (lowest panel). Scale bars: 50 µm. c) Kymograph along the line selections shown in b). Scale bars: 100 s and 20 µm. d) mCh-MTS constructs with two

copies of the *E. coli* MinD MTS exhibit a high contrast. Box plot of the contrast of mCh-MTS constructs, lines are median, box limits are quartiles 1 and 3, whiskers are 1.5 x IQR and points are outliers, generated from three independent experiments with in total *N*= 294 images per condition. Blue line marks no difference between the intensities in the minima and maxima of the MinDE wave (zero contrast). e) mCh-MTS intensity in the MinDE maximum ($\langle \boldsymbol{I} \rangle^{\text{mCh-MTS}}_{\text{max(MinD)}}$) normalized to His-mCh and corrected for the fluorescent protein fraction. f) Average EGFP-MinD intensity of the full image normalized to the fluorescence in the presence of His-mCh ($\langle \boldsymbol{I} \rangle^{\text{EGFP-MinD}}$). EGFP-MinD intensity, i.e. density, on the membrane is not influenced by the addition of mCh-MTS constructs. Each data point (squares, spheres, triangles) (exp1-3) was generated from 98 images in one sample chamber. Cross and error bars represent the mean value and standard deviation of the three independent experiments with in total *N*=294 images per condition.

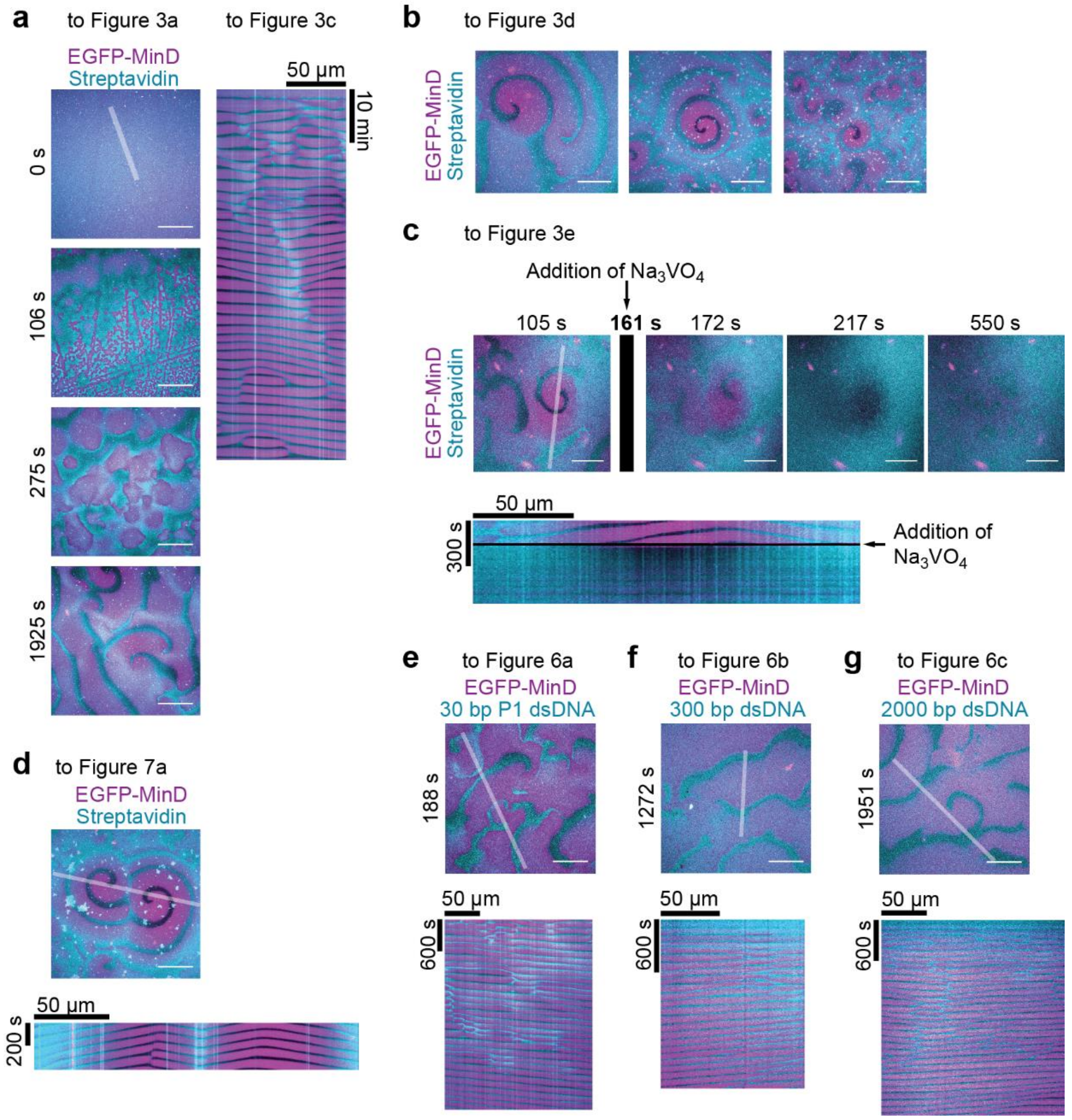

Supplementary Figure 8: Composite image of all separate channels shown in the main text involving streptavidin bound to biotinylated lipids. a) Composite image of MinDE self-organization (magenta) on a SLB with Biotinyl-CAP-PE-bound streptavidin (cyan) (1 µM MinD, 1 µM MinE, streptavidin-Alexa647) shown in Figure 3a. ATP is added at t=0 s to start self-organization. Scale bars: 50 µm. Kymograph along the line selection. b) MinDE self-organization leads to large scale concentration gradients of streptavidin. Representative composite images of streptavidin distribution in MinDE spirals after >1h of MinDE self-organization on SLBs as shown in Figure 3d. Scale bars 50 µm. c) Large scale streptavidin gradient formation by MinDE is reversible. Representative composite images and kymograph of a running MinDE assay in the presence of anchored streptavidin as shown in Figure 3e. Addition of sodium orthovanadate (Na_3VO_4) leads to MinDE detachment which in turn leads to homogenization of streptavidin fluorescence on the membrane. Scale bars: 50 µm. d) Streptavidin cannot dissociate in solution and is moved laterally on the membrane leading to accumulation on collision interfaces and depletion in spiral centers. Representative composite image of colliding MinDE waves in the presence of streptavidin bound to biotinylated lipids (1 µM MinD (30 % EGFP-MinD), 1 µM MinE, Alexa647-streptavidin) as shown in Figure 7a. Kymographs along the selection shown in the images. Scale bars: 50 µm. e) MinDE self-organization can regulate short membrane-anchored DNA fragments. Representative composite images and kymograph of a time-series of MinDE self-organization (magenta) in the presence of a 30 bp P1 dsDNA (cyan) bound to the membrane by a cholesterol anchor (1 µM MinD (30 % EGFP-MinD), 1 µM MinE, 10nM TEG-cholesterol-dsP1) shown in Figure 6a. Scale bars: 50 µm. f) Representative composite images and kymograph of a time-series of MinDE self-organization regulating 300 bp long dsDNA bound to lipid-anchored streptavidin (1 µM MinD (30 % EGFP-MinD), 1 µM MinE, 300 bp lambda DNA, streptavidin) shown in Figure 6b. Scale bars: 50 µm. g) Representative composite images and kymograph of a time-series of MinDE self-organization regulating 2000 bp long dsDNA bound to lipid-anchored streptavidin shown in Figure 6c. Scale bars: 50 µm.

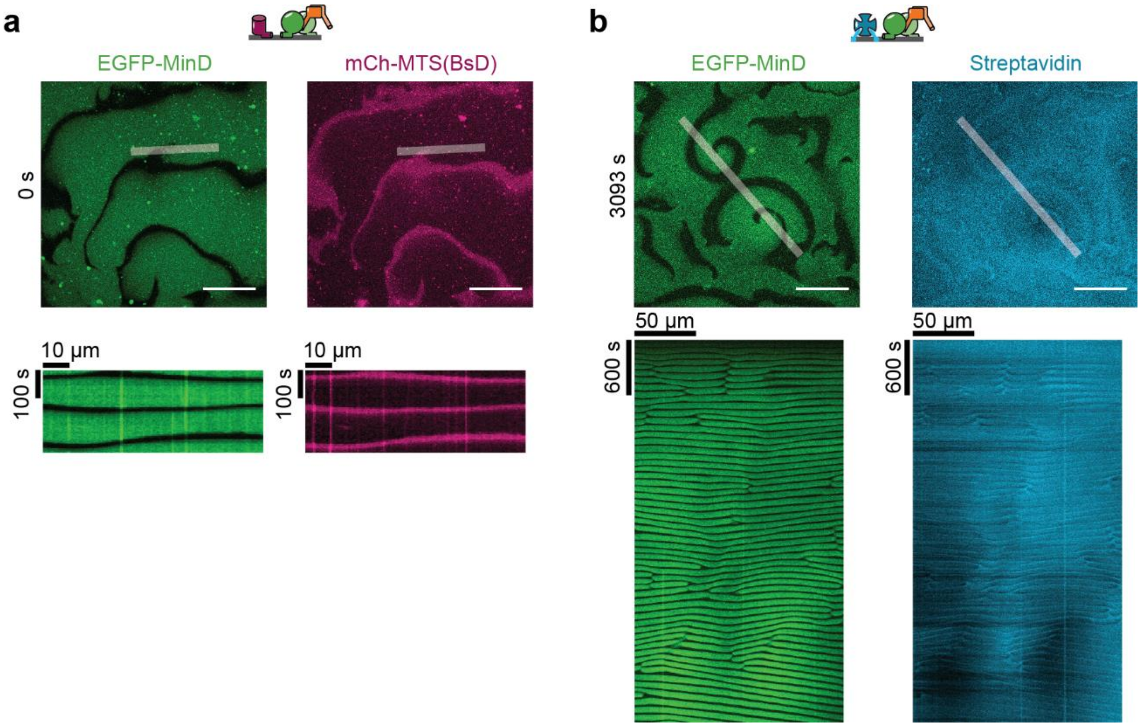

Supplementary Figure 9: MinDE position model membrane proteins on supported lipid bilayers made from *E. coli* polar lipid extract. a) Representative images and kymograph of the MinDE wave and the anticorrelated mCh-MTS(BsD) waves on SLBs made from *E. coli* polar lipid extract (1 µM MinD (30 % EGFP-MinD), 1 µM MinE, 1 µM mCh-MTS(BsD)). b) Representative images and kymograph of MinDE regulating Alexa647-streptavidin on SLBs made from *E. coli* polar lipid extract doped with 1 % Biotinyl-CAP-PE (1 µM MinD (30 % EGFP-MinD), 1 µM MinE, Alexa647-streptavidin). Experiments were performed independently three (b) or two (a) times under similar or identical conditions. Scale bars: 50 µm

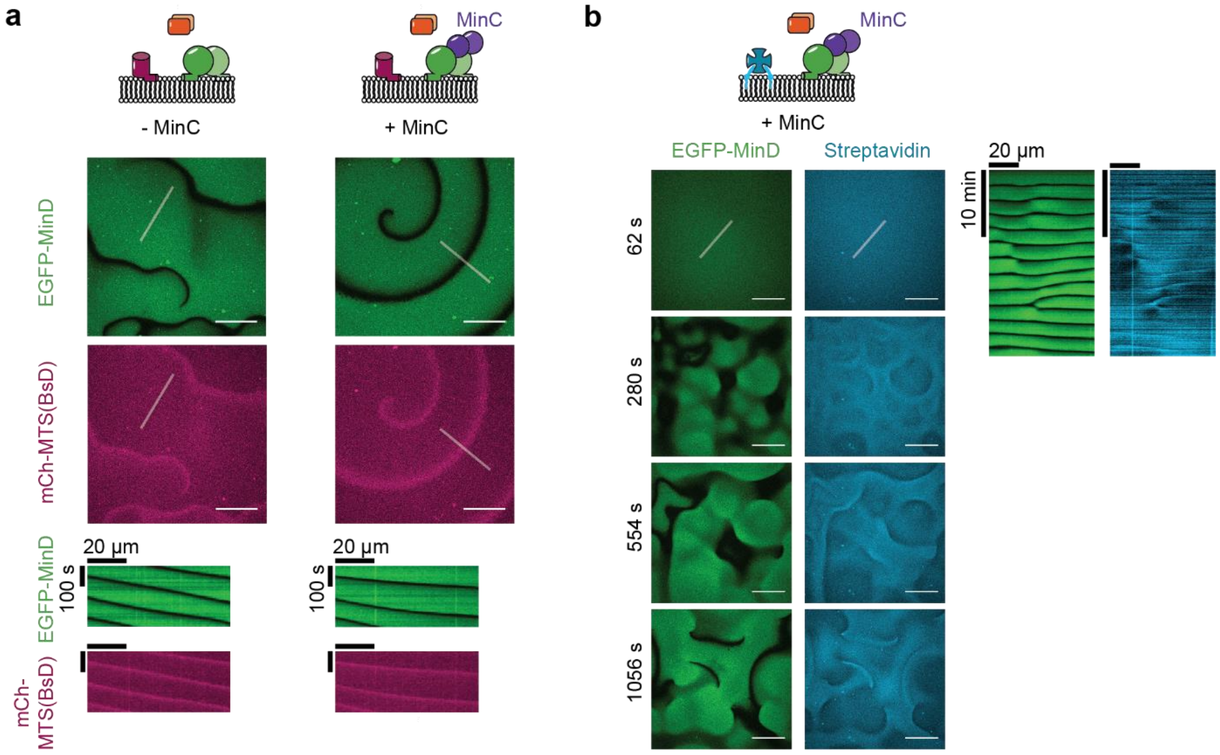

Supplementary Figure 10: Positioning of model membrane proteins by MinDE is independent of MinC. a) Representative images and kymograph of MinDE regulating mCh-MTS(BsD) in the presence or absence of MinC (1 µM MinD (30 % EGFP-MinD), 1 µM MinE, with and without 0.05 µM MinC, 1 µM mCh-MTS(BsD)) b) Representative images and kymograph of MinDE positioning lipid-anchored streptavidin in the presence of MinC (1µM MinD (30 % EGFP-MinD), 1 µM MinE, 0.05 µM MinC, Alexa647-streptavidin). Experiments were performed independently three (a) or two (b) times under identical conditions. Scale bars: 50 µm

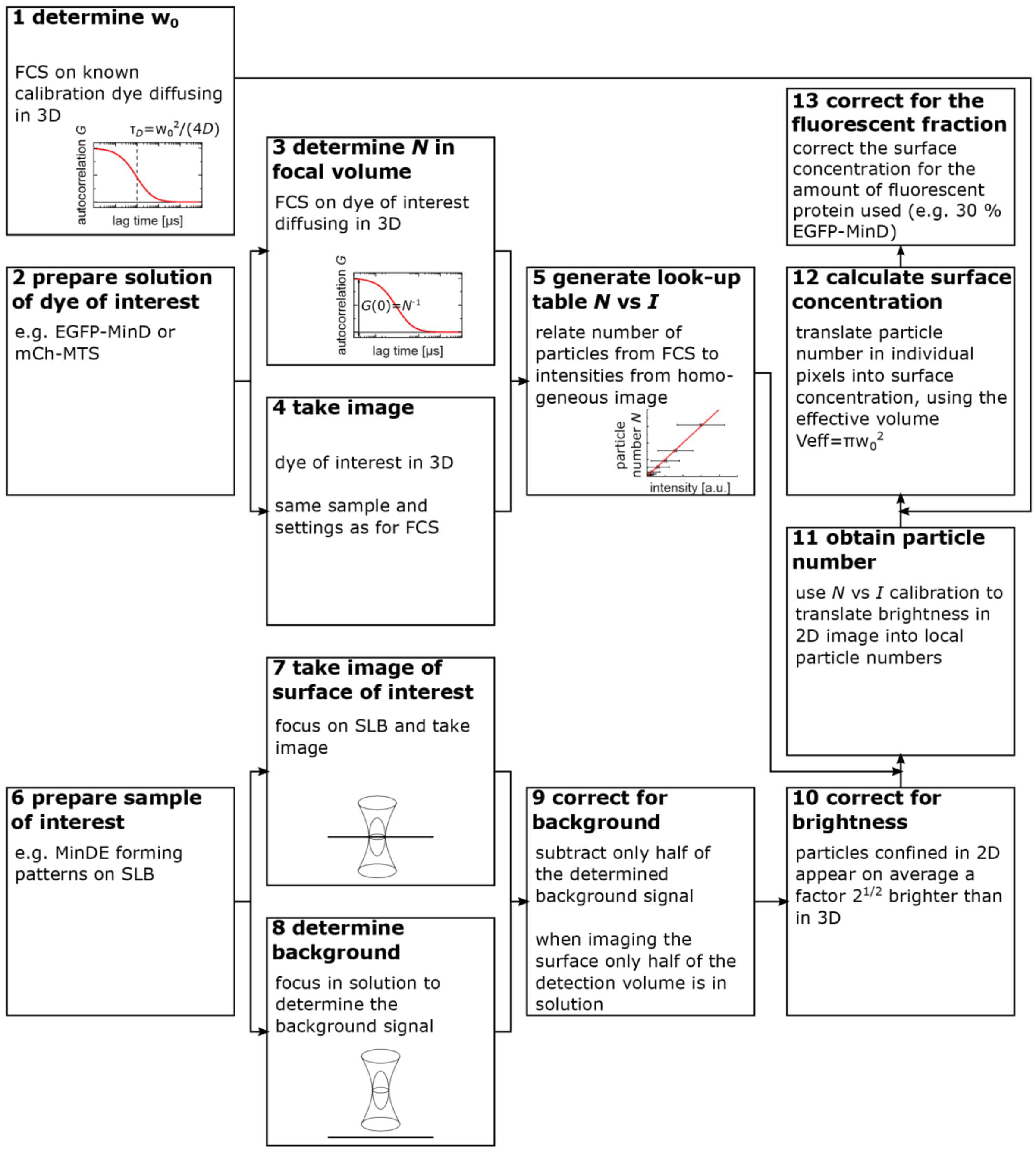

Supplementary Figure 11: Flowchart describing the Fluorescence Correlation Spectroscopy (FCS)-based image calibration performed to obtain EGFP-MinD and MTS(2xMreB)-mCh densities on the membrane.

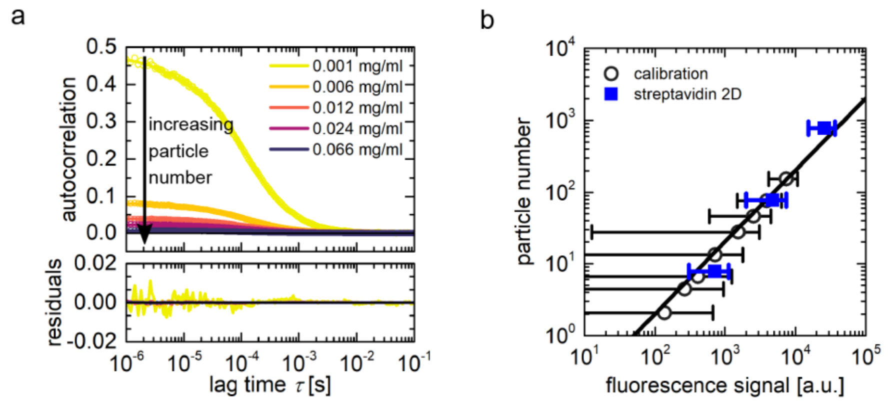

Supplementary Figure 12: Validation of the FCS-based quantification of surface densities. a) Representative autocorrelation curves for varying concentrations of streptavidin labeled with Alexa Fluor 488 freely diffusing in 3D. The experimental autocorrelation curves were fitted by a 3D diffusion model with a triplet contribution. b) The particle numbers obtained from the fit in a) are related to the corresponding fluorescence signal (black circles, mean±std) of a separately taken 100x100 pixel image (1 pixel corresponds to 420 nm). This data set is fitted by a line through the origin, yielding a calibration line, which relates the fluorescence signal in one pixel to the number of particles in the respective detection volume. Note the double-logarithmic scales. The linear relation adequately describes the experimental data over at least two orders of magnitude. The particle number $\boldsymbol{N}$ is easily translated into a 3D concentration via the relation $\boldsymbol{c_{3D}} = \boldsymbol{N}\left(\boldsymbol{\pi^{\frac{3}{2}} w_0^3 S}\right)^{-1}$, where $\boldsymbol{w_0}$ and $\boldsymbol{S}$ are obtained from the initial calibration experiment on freely diffusing dye (compare step 1 in Supplementary Fig. 11). To validate the approach outlined in Supplementary Fig. 11, we imaged SLBs containing varying fractions of biotinylated lipids, which recruit the fluorescently labelled streptavidin. From the molar fraction of these lipids, their density and the streptavidin-biotin valency[1] the mean number of particles in the detection volume can be estimated (here 7.8, 78, and 778 particles per detection volume). A 100x100 pixel image (1 pixel corresponds to 420 nm) of the SLB was taken, and the pixel values divided by $\sqrt{\mathbf{2}}$ (compare Supplementary Fig. S11). The corresponding pairs of fluorescence signal (mean±std) and the expected particle number (blue squares) were superimposed on the calibration curve, showing good agreement, and thus validate the proposed approach.

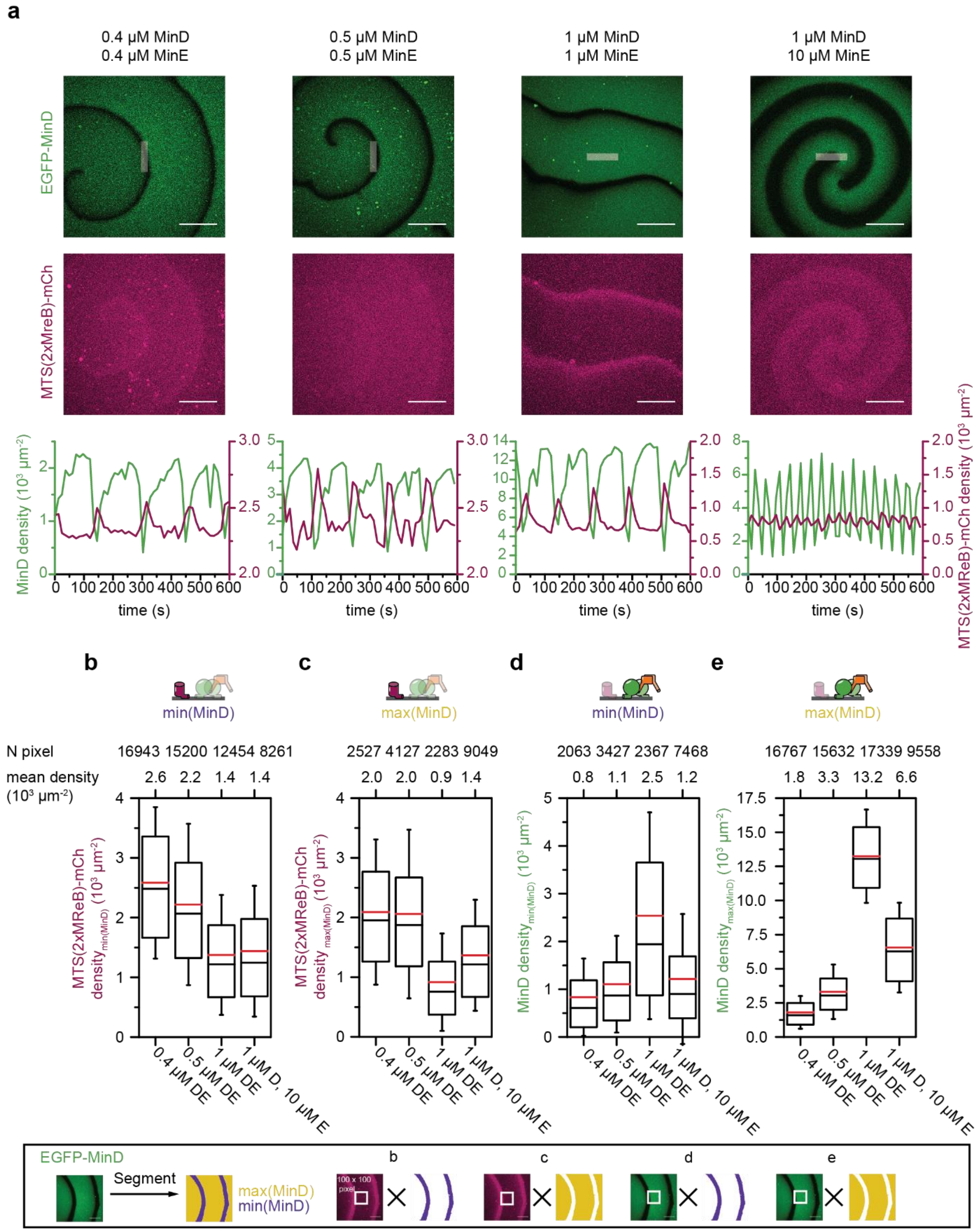

Supplementary Figure 13: MinDE waves spatiotemporally regulate MTS(2xMreB)-mCh at MinD densities on the order of magnitude occurring *in vivo*. a) Representative images used for density quantification (upper panels), temporal density profiles along the 20 by 100 pixel selection shown in the images. Scale bars: 50 µm b) Boxplots of density values in a 100 by 100 pixel region in the center of all images for b) MTS(2xMreB)-mCh in the MinD minimum, c) MTS(2xMreB)-mCh in the MinD maximum, d) MinD in the MinD minimum, e) MinD in the MinD maximum. Box limits are quartiles 1 and 3 and whiskers are standard deviation, median is shown as a black line, mean is shown as a red line. Data from two independent FCS/image calibrations.

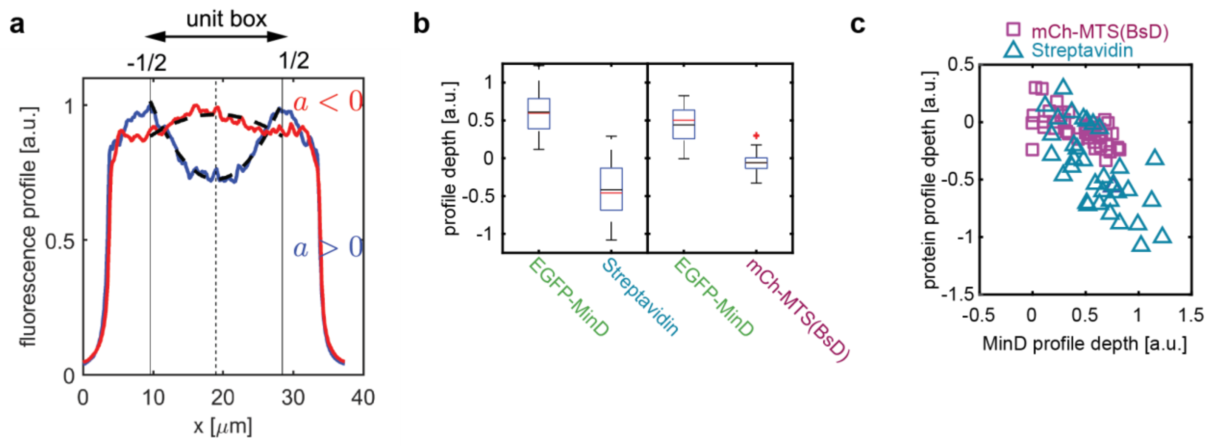

Supplementary Figure 14: Quantification of time-averaged fluorescence intensity profiles. a) Normalized, time-averaged fluorescence intensity profiles of EGFP-MinD (blue) and streptavidin (red). Vertical lines indicate the maxima of the EGFP-MinD profile that define the edges of the unit box, i.e. a box of length 1. Thus, every profile is projected from its physical extent onto the unit box, ranging from -1/2 to 1/2. Dashed lines indicate the quadratic function fit to the profiles in the unit box to determine the profile depth. The curvature a is a direct measure for the depth of the profile. b) Box plot of the depth of the fluorescence intensity profile for mCh-MTS(BsD) and streptavidin with the respective EGFP-MinD one. Box limits are quartiles 1 and 3 and whiskers are 1.5 x IQR, median is shown as a black line, mean is shown as a red line, points are outliers. (Data from three independent experiments with mCh-MTS(BsD) = 45 and streptavidin = 35 individual microcompartments). c) Depths of individual fluorescence intensity profiles of mCh-MTS(BsD) and streptavidin plotted against the depth of the EGFP-MinD fluorescence intensity profile. mCh-MTS(BsD) profile depth is close to zero independent of EGFP-MinD profile depth. Streptavidin profile depth of individual fluorescence intensity profiles is correlated with EGFP-MinD profile depth, indicating a strong regulation of membrane-anchored proteins by MinDE.

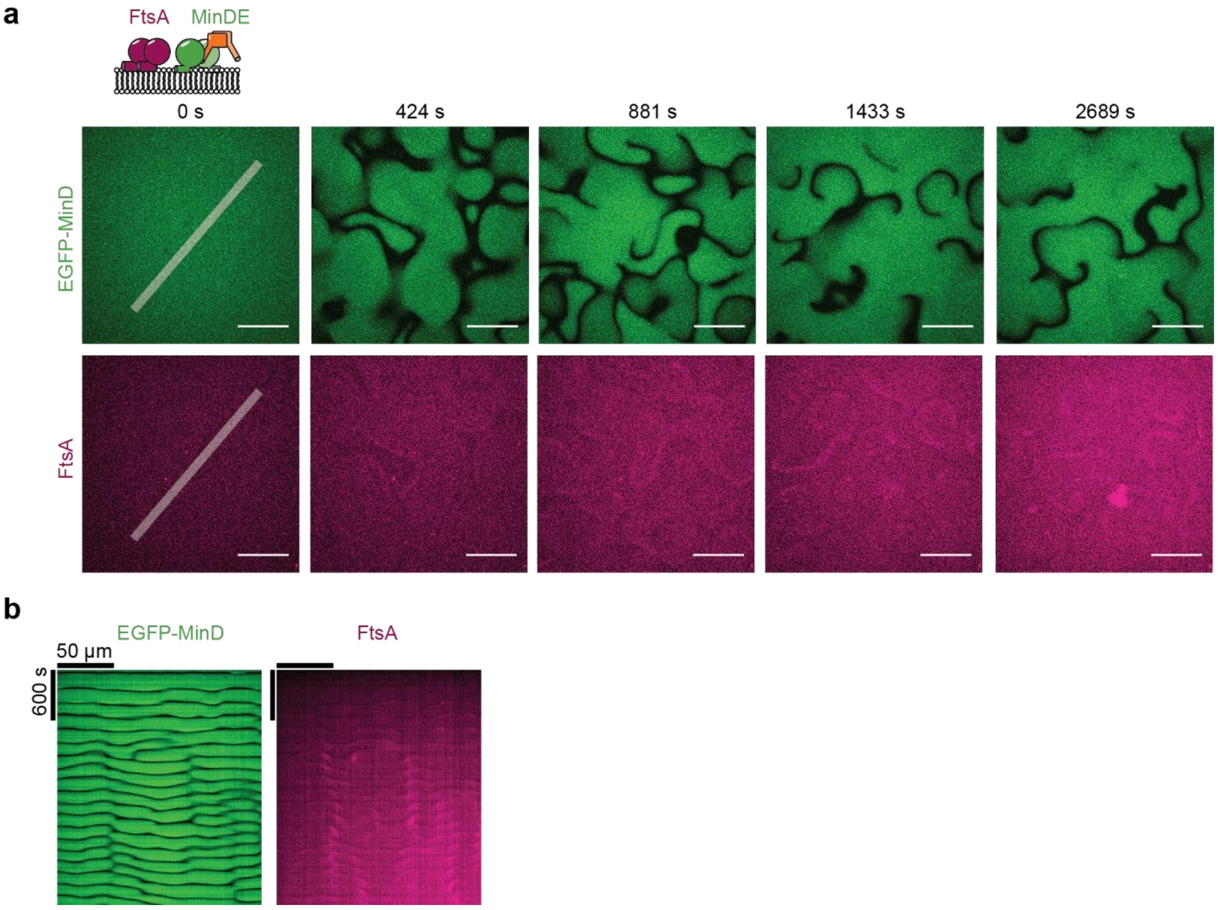

Supplementary Figure 15: MinDE spatiotemporally regulates FtsA. a) Representative images of time series of MinDE self-organization in the presence of FtsA (0.4 µM Cy5-FtsA, 1 µM MinD (30 % EGFP-MinD), 1 µM MinE). ATP is added at t=0 s to start self-organization. Scale bars: 50 µm. b) Kymograph along the line selection shown in a). Experiment was performed independently two times under similar or identical conditions. Scale bars: 50 µm and 600 s

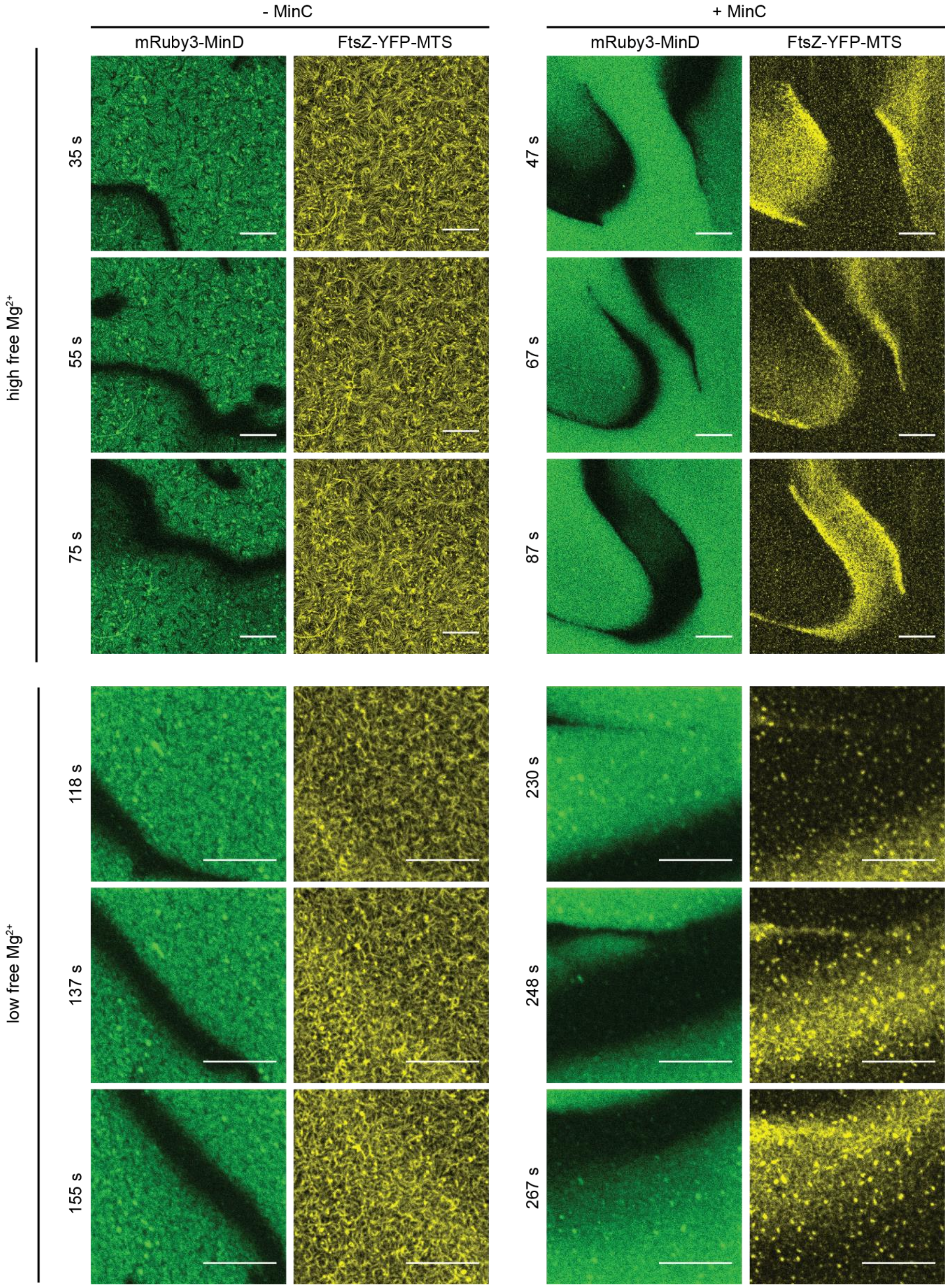

Supplementary Figure 16: MinDE cannot move filaments and dynamic rings of FtsZ-YFP-MTS laterally. Representative images of MinDE self-organization in the presence of FtsZ-YFP-MTS with high and low free Mg^{2+} and with and without MinC (1 µM MinD (30 % EGFP-MinD), 1 µM MinE, 0.5 µM FtsZ-YFP-MTS, with and without 0.05 µM MinC) at high magnification. Brightness/contrast settings are not comparable between images. For low Mg^{2+} conditions images were acquired with a line average of 4 for better resolution. Scale bars: 10 µm

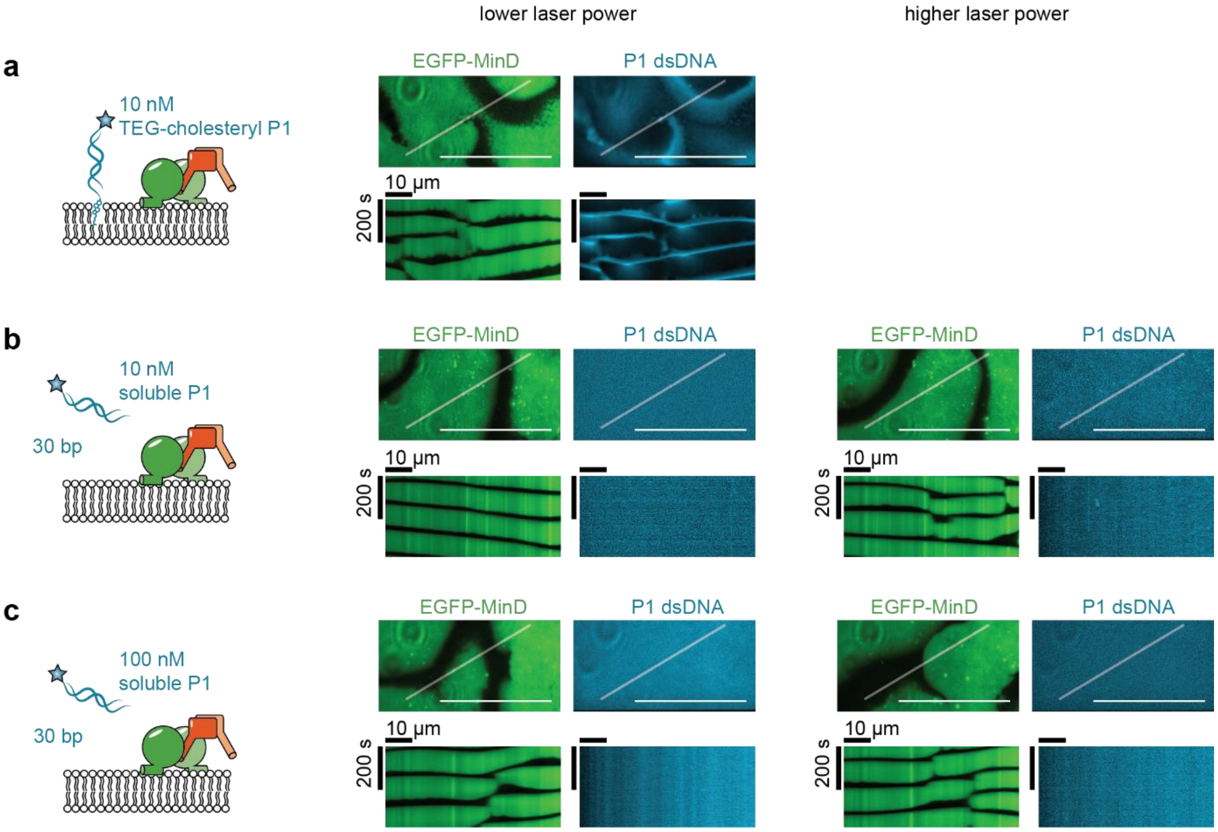

Supplementary Figure 17: Binding of soluble P1 dsDNA fragment to MinD cannot be observed in TIRF. a) Spatiotemporal positioning of TEG-cholesterol anchored P1 DNA fragment by MinDE is clearly visible in TIRF microscopy images (1 µM MinD (30 % EGFP-MinD), 1 µM MinE, 10 nM TEG-cholesterol dsP1). No modulation in the fluorescence intensity signal of soluble P1 dsDNA fragment can be observed for b) 10 nM or c) 100 nM P1 dsDNA, when added to MinDE self-organization assays (1 µM MinD (30 %E GFP-MinD), 1 µM MinE, 10 or 100 nM soluble P1). Images were acquired at two different laser powers. Experiments were performed independently two times under identical conditions. Scale bars: 50 µm

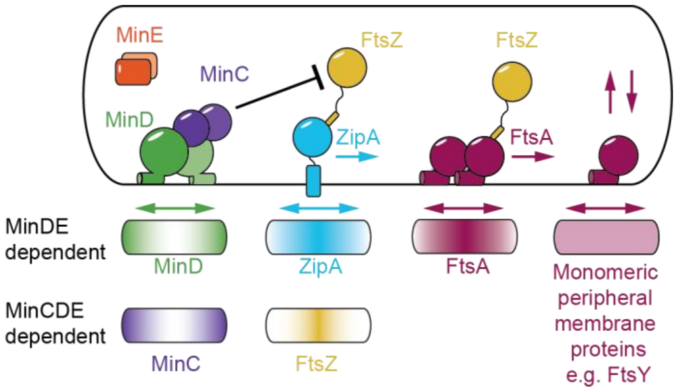

Supplementary Figure 18: Schematic model of how the MinCDE system could influence positioning of major divisome proteins FtsZ, ZipA and FtsA. MinC, antagonist of FtsZ assembly, whose time-averaged concentration is highest at the cell poles confines FtsZ polymerization to midcell. MinDE oscillations might establish a time-averaged concentration gradient of the transmembrane protein ZipA and the oligomerized FtsA that is maximal at midcell. MinDE might establish counter-oscillations of monomeric peripheral membrane proteins decreasing protein abundance on the membrane.

Supplementary Table 1

List of observations possibly linked to MinDE-mediated regulation of membrane proteins

Possible target	Observation	study
FtsY, UspE, PfkA, Pgk, YciG	Abundance of peripheral membrane proteins is decreased in the presence of MinCDE as compared to a Δ*minCDE* strain	[2]
ZipA	ZipA and MinCDE counteroscillations; no counteroscillation in a Δ*minCDE* strain	[3]
ZipA	ZipA although essential in *E. coli* can be bypassed by mutations in other divisome proteins and thus has been suggested to enhance cell division.	[4–7]
Differential regulation of FtsA, FtsA*	FtsA mutant (FtsA*) impaired for self-interaction can bypass ZipA. Monomeric FtsA has been suggested to recruit other divisome proteins.	[6,7]
FtsZ anchors, ZipA and FtsA	Slow FtsZ oscillations on the timescale of MinDE oscillations; no detectable slow FtsZ oscillations in a Δ*minCDE* strain	[8]
FtsZ anchors, ZipA and FtsA	Similar FtsZ dynamics for WT and Δ*minC* strains, but altered dynamics in a Δ*minCDE* strain	[9]
B. subtilis membrane-bound division proteins	*E. coli* MinDE oscillate in *B. subtilis* and impair sporulation by inhibiting polar septum formation in the absence of both *E.coli* and *B. subtilis* MinC	[10]
TnaA, GroES, YqjD	Polar localization of three foci-forming inner membrane associated proteins is disrupted in Δ*minCDE* strain	[11]
chromosome	Abnormal nucleoid distribution and anucleate rod-shaped cells in MinCDE mutants	[12–14]
chromosome	Strains deleted for the histone-like protein HU acquire secondary mutations in *minCDE*	[15]
chromosome	Overexpression of MinE impairs nucleoid segregation	[16]
chromosome	Disturbed chromosome segregation in Δ*minCDE* strains	[17]

Supplementary Table 2

List of plasmids

Vector	Protein name	Reference or Source
pET28a-His-MinD_MinE	MinD	[18]
pET28a-His-EGFP-MinD	EGFP-MinD	[19]
pET28a-His-MinE	MinE	[18]
pML60	Sumo-Gly5-FtsA	[20]
pLVX-mCherry-C1		Clontech
pCoofy1-mCherry	His-mCh	Lei Kai
pET28a-BsMTS-mCherry-His	BsMTS-mCherry	This study
pET28a-mCherry-GGBsMTS	mCh-MTS(BsD)	This study
pET28a-MreBN-mCherry-His	MTS(1xMreB)-mCh	This study
pET28a-2xMreBN-mCherry-His	MTS(2xMreB)-mCh	This study
pET28a-mCherry_FtsA	mCh-MTS(FtsA)	This study
pET28a-FtsY_mCherry	MTS(FtsY)-mCh	This study
pET28a-mCherry_EcMTS	mCh-MTS(1xMinD)	This study
pET28a-mCherry_2xEcMTS	mCh-MTS(2xMinD)	This study
pET28a-mCherry-Jun-EcMTS	mCh-Jun-MTS(1xMinD)	This study
pET28a-mRuby3-MinD	mRuby3-MinD	[21]
pET11b-FtsZ-YFP-MTS	FtsZ-YFP-MTS	[22]

Supplementary Table 3

List of primers

**Primername**	**Sequence 5' to 3'**
21_ YFP_for	ATGGTGAGCAAGGGCGAG
22_ YFP_rev	CTTGTACAGCTCGTCCATGC
23_ YFP_MinD_for	CATGGACGAGCTGTACAAGGAATTCGCACGCATTATTGTTG
24_ pET_YD_rev	CTCGCCCTTGCTCACCATGGATCCGCGACCCATTTG
64_BSMTS-mCH_fw	AATCAAATCTTTCTTCGGTTCTTCTGGTTCTTCTGGTATGGTGAGCAAGG GCGAG
65_BSMTS-pET_rev	GAACCGAAGAAAGATTTGATTTTAGCCATCATACCTTTACTGCTGCCCAT GGTATATCT
68_His-mcherry_rev	TGGTGGTGGTGGTGCTCGACTCCAGATCCACCCTTGTACAGCTCGTCCAT G
pET28a_fw	GTCGAGCACCACCACCA
19_mCherry_fw	TCAGGTGGAAGTCCTAGGATGGTGAGCAAGGGCGAG
20_pET28a_rev	CCTAGGACTTCCACCTGAA
50_BsMTS-pET	GTATGATGGCTAAAATCAAATCTTTCTTCGGTGTTCGTTCTTAAAAGCTT GCGGCCGCA
61_BsMTS_mCh_rev	ATTTGATTTTAGCCATCATACCTTTGCCAGATCCCTTGTACAGCTCGTCCA TGCC
80_MreBN-mCherry_fw	AAATTCCGTGGTATGTTCCGTGGTTCTGGTTCTTCTGGTTCTTCTGGT
81_MreBN-pET_rev	ACGGAACATACCACGGAATTTTTTCAGCATGGTATATCTCCTTCTTAAAG TTAAA
78_MreBN2x-pEt28a_rev	CAGAACCACCGAACATACCACGGAATTTTTTCAGCATGGTATATCTCCTT CTTAAAGTTAAA

79_MreBN2x-mCherry_fw	AACAGCAGCTGAAAAAATTCCGTGGTATGTTCCGTGGTTCTGGTTCTTCT GGTTCTTCTGGT
BR200_mCherry_FtsA_for	ATTGAGTCGCTTGATCCACGAGCCGCCAGATCCCTTGTACAGC
p11_MTSftsA_rev	AGTTGGCTGCGAAAAGAGTTTTAAGGATCCGGCTGCTAACAAAG
BR201_fw_FtsY_mCherry	CCTGTTAAAAACCAAAGAAAATCTCGGTCGTGGTTCTGGTTCTTCTGGTT
BR202_FtsY_mCherry_rev	CTGCGTTTCAGGCGCGCGAACATGGTATATCTCCTTCTTAAAGTTAAAC
BR234_tandemMinD_fw	TAAAAGCTTGCGGCCGCACTC
BR235_tandemMinD_rev	TCCTCCGAACAAGCGTTTGAGGAAGCCTTTCTTCTCTTCTTCAATagatccg ccTCCTCCGAACAAGCGTTTGAGGA
43_mut_Kan_fw	TGAAACATGGCAAAGGTAGCGT
93_mCh-EcMTS_fw	AGCTGTACAAGGGATCTGGCATTGAAGAAGAGAAGAAAGGCTTCCTC
94_GSG_mCh_rev	GCCAGATCCCTTGTACAGCTCG
44_mut_Kan_rev	GCTACCTTTGCCATGTTTCAGAAA
472_Lin-RV	GCCAGATCCCTTGTACAGC
473_Lin-FW	ATTGAAGAAGAGAAGAAAGGC
474_Jun-FW	CTGTACAAGGGATCTGGCCGTATCGCTCGTCTGGAA
475_Jun-RV	TTTCTTCTCTTCTTCAATAGAACCTCCTCCACCGTAGTTCATAAC
LP2_fw	CGCCATTAACCTGATGTTCTGGGG
FW_P1_30bp_chol	GAATCAGCGCCATTTATCACAGAATAGACT-CHOLESTEROL
FW_P1_30bp_sol	GAATCAGCGCCATTTATCACAGAATAGACT
RV_P1_30bp_Al647	AGTCTATTCTGTGATAAATGGCGCTGATTC-ALEXA647
BR215_Cy5_tetO_lambda_fw	AGACATCCCTATCAGTGATAGAGACGCGGGTTTTCGCTATTTATGAAAAT TTTC
BR120_5'BiotinTEG_l300_rev	TAAAGCACCTCATTACCCTTGCCAC
BR122_5'BiotinTEG_l2000rev	ATCGTCGTGGCGGCC

Supplementary Note 1

In vivo MinDE densities. MinD and MinE concentrations in *E. coli* have been determined to be about 2000-3000 molecules per cell by western-blotting[23,24]. Assuming that almost all proteins bind to the membrane and do so only at one pole at a time, and an average inner membrane area of about 6 μm^2, the density of MinD on the membrane can be estimated to about $1 \times 10^3\ \mu m^{-2}$. Furthermore, a large fraction of the total membrane surface in vivo is occupied (~60%) by transmembrane proteins[25], further reducing the available membrane area and potentially increasing the local MinDE density.

Supplementary Note 2

MinDE change the physical properties of membranes. The MTS of both MinD and MinE are amphipathic helices, known to change the physical properties of membranes[26]. They insert into the membrane leading to deformation of liposomes[27–29], change the membrane viscosity in vitro[30], preferentially bind to anionic lipids[31–33] and can stabilize lipid domains[34]. Interestingly, stains for membrane properties, such as cardiolipin density, resemble the MinD localization at cell poles and nascent septa[35,36], which led to the suggestion that MinDE self-organization relies on certain lipid distributions as a spatial cue[37]. However, this hypothesis has been disproved by showing that geometry sensing is an intrinsic property of the MinDE system[31,38–40].

Supplementary Methods

Estimation of protein surface densities

We estimated the surface concentration from confocal fluorescence images based on a multistep procedure, as outlined in Supplementary Fig. 11. In essence, this approach relies on a combined confocal Fluorescence Correlation Spectroscopy (FCS)[41–43] and imaging-based calibration, which relates the pixel values to the number of particles in this pixel. Similar approaches have been introduced before. [44–46]

In an initial step (box 1 in Supplementary Fig. 11), the effective detection volume $V_{\mathrm{eff}} = \pi^{\frac{3}{2}} w_0^3 S$ was calibrated based on the diffusion coefficient of a known dye, which has spectral properties similar to the fluorescent tag whose density needs to be determined later. Here, w_0 is the lateral width of the Gaussian detection volume $\Omega(x,y,z) = \Omega_0 \exp\left(-2\frac{x^2+y^2}{w_0^2}\right)\exp\left(-2\frac{z^2}{(Sw_0)^2}\right)$ and S is the structure parameter, which describe the elongation of this volume. In this work, ATTO488-COOH and Alexa Fluor 546-NHS freely diffusing in water were used to calibrate the detection volumes for measurements on EGFP and mCherry, respectively. The corresponding diffusion coefficients at 25°C are D = 405 $\mu m^2\ s^{-1}$ for ATTO488 (unpublished data) and D = 364 $\mu m^2\ s^{-1}$ for Alexa Fluor 546.[47] The temperature at the objective was monitored by a thermocouple and the diffusion coefficient was corrected for the respective temperature T using the Stokes-Einstein-Smoluchowski relation $D \propto T/\eta(T)$.[48–50] The acquired autocorrelation curves were fitted by a model function for combined 3D diffusion and blinking:

$$G(\tau) = N^{-1}\left(1 + \frac{f}{1-f}\exp(-\frac{\tau}{\tau_T})\right)\left(1 + \frac{\tau}{\tau_D}\right)^{-1}\left(1 + \frac{\tau}{S^2\tau_D}\right)^{-\frac{1}{2}} \quad (1)$$

Here, f is the fraction of molecules in a dark state, e.g. triplet, and τ_T is the associated characteristic decay time. As $\tau_D = \frac{w_0^2}{4D}$ and S are obtained from the fit, V_{eff} can be readily calculated. Moreover, the number of fluorescent particles N in the detection volume is obtained. This calibration was performed on a daily basis and for each spectral channel separately.

Next (boxes 2-5 in Supplementary Fig. 11), a relation between image brightness and the number of particles in one pixel, which corresponds to one detection volume, was determined. Therefore, a solution of the protein of interest was prepared, e.g. EGFP-MinD or mCh-MTS above a non-charged supported lipid bilayer (100 mol % DOPC). In a first step, another FCS measurement in solution was performed. From the autocorrelation curve and its fit (eq. 1), the number of particles N was obtained and corrected for uncorrelated background.[51] Subsequently, a fluorescence image was taken in solution and background corrected. Thus, the mean signal I in this image was connected to a number of particles N. This approach was repeated for several concentrations to map the relation N vs. I, which was fitted by a linear function (compare Supplementary Fig. 11). Importantly, the imaging settings were identical for the FCS measurements, the corresponding imaging and all subsequent images, which relied on the determined relation N vs. I.

To estimate surface concentrations, the sample of interest, e.g. MinDE forming patterns on an SLB, was prepared and imaged with the membrane being in focus. To determine the effective background of fluorescently labelled protein that was not membrane bound, we took another image in solution far above the membrane. For a simplified system with no axial concentration gradient, the signal measured in solution corresponds to twice the background signal of an image taken on the membrane, because in this scenario, only the upper half of the detection volume collects fluorescence from the solution. The resulting pixel values cannot directly be translated into particle numbers via the previously determined relation N vs. I, because particles that are on the membrane have a higher average apparent brightness $\bar{Q}$ than identical particles diffusing in 3D. This effect results from the restriction to two dimensions, which implies that all particles are always perfectly in focus, i.e. in the axial maximum of the 3D Gaussian Ω. Consequently, the average apparent brightness $\bar{Q}$ in 2D and 3D reads:

$$\bar{Q}_{\mathrm{2D}} \propto \frac{1}{V_{\mathrm{eff}}} \int dx \int dy \exp\left(-2\frac{x^2+y^2}{w_0^2}\right) \exp\left(-2\frac{z^2}{(Sw_0)^2}\right)$$

$$\bar{Q}_{\mathrm{3D}} \propto \frac{1}{V_{\mathrm{eff}}} \int dx \int dy \int dz \exp\left(-2\frac{x^2+y^2}{w_0^2}\right) \exp\left(-2\frac{z^2}{(Sw_0)^2}\right)$$

$$\frac{\bar{Q}_{\mathrm{2D}}}{\bar{Q}_{\mathrm{3D}}} = \sqrt{2}$$

Thus, the membrane-bound particles appear a factor of $\sqrt{2}$ brighter than in solution. After correction for this factor, the previously determined relation N vs. I was applied to determine the particle number in each pixel. Since w_0 was initially determined, the obtained N in each pixel can be converted into a surface concentration $C = \frac{N}{\pi w_0^2}$. Further, this surface concentration was corrected for the fluorescent fraction of proteins used (box 13 Supplementary Fig. 11). To validate this approach, we determined Alexa488-streptavidin densities bound to SLBs containing different amounts of biotinylated lipids (70 mol % DOPC, 30 mol % DOPG, 0.01/0.1/1 mol % Biotinyl-CAP-PE), prepared in the same way as described in the methods section (Supplementary Fig. 12). To determine the densities of MTS(2xMreB)-mCh and EGFP-MinD on the membrane, the images were first calibrated as described above. We further generated the binary masks of the MinDE wave from the original EGFP-MinD as described (Analysis of mean fluorescence intensities in MinDE wave minima and maxima). The calibrated images were multiplied with the binary masks and all zero values were removed. All pixels located in a centered 100 x 100 pixel region from all images of one condition were pooled to obtain the box plot and average protein densities on the membrane in the MinDE minima ((MTS(2xMreB)-mCh density$_{\mathrm{min(MinD)}}$/MinD density$_{\mathrm{min(MinD)}}$) and maxima ((MTS(2xMreB)-mCh density$_{\mathrm{max(MinD)}}$/MinD density$_{\mathrm{max(MinD)}}$).

Cloning methods

For cloning all plasmids were propagated in *E. coli* OneShot TOP10 (Invitrogen, Thermo Fisher Scientific, Waltham, USA). We used seamless assembly for the cloning of larger DNA fragments into vectors. DNA fragments and vector backbones were amplified by PCR with primers that contained 15-

20 bp overlaps between adjacent fragments. The PCR products were then combined using GeneArt Seamless Cloning and Assembly Enzyme Mix (Thermo Fisher Scientific, Waltham, USA) according to manufacturer's instruction.

We used blunt end cloning for the introduction of point mutations or small peptide sequences. The entire vector was amplified with two primers extended by the sequence to be introduced. After PCR the product was digested with DpnI to remove plasmid template. The blunt ends of the PCR products were phosphorylated using T4 Phosphokinase and subsequently ligated with T4 DNA Ligase (Thermo Fisher Scientific).

The plasmids for the purification of His-MinD[18], His-EGFP-MinD[19], His-MinE[18] and FtsA (pML60)[20] have been described previously.

pET28a-BsMTS-mCherry-His encodes the N-terminal fusion of the Bacillus subtilis MTS (KGMMAKIKSFFGSSGSSG (AA 254-265 of *B.subtilis* MinD) to mCherry and a C-terminal His-Tag. The coding region for mCherry was amplified by PCR from the plasmid pLVX-mCherry-C1 (Clontech, Mountain View, CA, USA) using the primers 64_BSMTS-mCH_fw/68_His-mcherry_rev and the backbone was amplified from pET28a using the primers pET28a_fw/65_BSMTS-pET_rev. The two PCR products overlap and were assembled using seamless assembly. pET28a-mCherry-GGBsMTS encodes for the C-terminal fusion of the *B. subtilis* MinD MTS (GSGKGMMAKIKSFFGVRS; AA 254-268 of *B. subtilis* MinD) to mCherry and an N-terminal His-Tag (mCh-MTS(BsD)). mCherry coding region was amplified from pLVX-mCherry-C1 (Clontech) using primers 19_mCherry_fw/61_BsMTS_mCh_rev and the backbone was amplified from pET28a with the primers 20_pET28a_rev/50_BsMTS-pET. PCR products were combined using seamless assembly. pET28a-MreBN-mCherry-His encodes for the N-terminal fusion of the *E. coli* MreB MTS (MLKKFRGMFRGSGSSGSSG; AA 1-9 of *E. coli* MreB) of mCherry with a C-terminal His-Tag (MTS(1xMreB)-mCh). The construct is designed analogously as the MreB-MTS fusion of EGFP in pFE356[52]. mCherry coding region was amplified from pET28a-BsMTS-mCherry-His with primers 68_His-mcherry_rev/80_MreBN-mCherry_fw and the backbone was amplified from

pET28a with primers pET28a_fw/81_MreBN-pET_rev. Both PCR products were subsequently combined using Seamless Assembly. pET28a-2xMreBN-mCherry-His encodes for an N-terminal fusion of two copies of the *E. coli* MreB MTS (MLKKFRGMFGGSEQQLKKFRGMFRGSGSSGSSG) to mCherry with a C-terminal His-Tag (MTS(2xMreB)-mCh). The construct was analogously constructed to the 2xMreB-MTS fusion of EGFP in plasmid pJS111.[52] The plasmid was amplified from pET28a-MreBN-mCherry-His with primers 78_MreBN2x-pEt28a_rev/79_MreBN2x-mCherry_fw and ligated using blunt end cloning. pET28a_mCherry_FtsA codes for a C-terminal fusion of the FtsA MTS to mCherry with an N-terminal His-Tag (mCh-MTS(FtsA)) (GSGGSWIKRLNSWLRKEF; AA 406-420 of *E. coli* FtsA) analogously to the GFP-FtsA-MTS fusion in plasmid pSEB295.[53] The mCherry coding regions from plasmid pET28a-mCherry-GGBsMTS were amplified with primers BR200_mCherry_FtsA_for/p11_MTSftsA_revA and subsequently religated using blunt end cloning. pET28a_FtsY_mCherry encodes the N-terminal fusion of the FtsY-MTS to mCherry with a C-terminal His-Tag (MTS(FtsY)-mCh) (MFARLKRSLLKTKENLGRGSGSSGSSG; AA 196-211 of *E. coli* FtsY) and was designed analogously to the NG+1-Trx fusion.[54] It was constructed by amplification of the mCherry coding region from pET28a-MreBN-mCherry-His using primers BR201_fw_FtsY_mCherry/BR202_FtsY_mCherry_rev and recombined with blunt end cloning. pET28a-mRuby3-MinD was cloned from a sequence-optimized, custom-ordered mRuby3[55] DNA fragment that was inserted in place of EGFP on pET28a-EGFP-MinD using primers 21_ YFP_for, 22_ YFP_rev, 23_ YFP_MinD_for and 24_ pET_YD_rev and Seamless Assembly. pET28a_mCherry_EcMTS codes for a C-terminal fusion of the *E. coli* MinD MTS to mCherry (mCh-MTS(1xMinD)) with an N-terminal His-tag (GSGIEEEKKGFLKRLFGG; AA 256 – 270 of *E. coli* MinD) analogously to the fusion to GFP in plasmid in pTS14[56]. It was constructed by amplification of the mCherry coding region from plasmid pET28a-mCherry-GGBsMTS using primers 94_GSG_mCh_rev/ 44_mut_Kan_rev and of the *E. coli* MTS sequence from plasmid pET28a-EGFP-MinD using primers 43_mut_Kan_fw/93_mCh-EcMTS_and assembly using seamless assembly.

pET28a_mCherry_2xEcMTS codes for a C-terminal fusion of two copies of the *E. coli* MinD MTS to mCherry (mCh-MTS(2xMinD)) with an N-terminal His-tag

(GSGIEEEKKGFLKRLFGGGGSIEEEKKGFLKRLFGG; AA 256 – 270 of *E. coli* MinD) analogously to the fusion to GFP in plasmid in pSLR92[56]. It was constructed by amplification of the mCherry coding region from pET28a_mCherry_EcMTS using primers BR234_tandemMinD_fw/BR235_tandemMinD_rev and recombined using blunt end cloning. pET28a-mCherry-Jun-EcMTS encodes for C-terminal fusion of the *E. coli* MinD MTS to an mCherry Jun leucine zipper fusion construct (mCh-Jun-MTS(1xMinD)) with an N-terminal His-tag (GGGGSIEEEKKGFLKRLFGG; AA 256 – 270 of *E. coli* MinD) analogously to pTS37[56]. It was constructed by amplification of the mCherry coding region from plasmid pET28a_mCherry_EcMTS using primers 472_Lin-RV/473_Lin-FW and the coding region Jun from a custom ordered gene sequence using primers 474_Jun-FW/475_Jun-RV that were combined using seamless assembly.

Supplementary References

1. Dubacheva, G. V. *et al.* Controlling Multivalent Binding through Surface Chemistry: Model Study on Streptavidin. *J. Am. Chem. Soc.* **139,** 4157–4167 (2017).

2. Lee, H.-L. *et al.* Quantitative proteomics analysis reveals the Min system of Escherichia coli modulates reversible protein association with the inner membrane. *Mol. Cell. Proteomics* **15,** 1572–1583 (2016).

3. Bisicchia, P., Arumugam, S., Schwille, P. & Sherratt, D. MinC, MinD, and MinE drive counter-oscillation of early-cell-division proteins prior to Escherichia coli septum formation. *MBio* **4,** e00856-13 (2013).

4. Hale, C. A. & De Boer, P. A. J. Direct binding of FtsZ to ZipA, an essential component of the septal ring structure that mediates cell division in E. coli. *Cell* **88,** 175–185 (1997).

5. Geissler, B., Elraheb, D. & Margolin, W. A gain-of-function mutation in ftsA bypasses the requirement for the essential cell division gene zipA in Escherichia coli. *Proc. Natl. Acad. Sci. U. S. A.* **100,** 4197–4202 (2003).

6. Pichoff, S., Shen, B., Sullivan, B. & Lutkenhaus, J. FtsA mutants impaired for self-interaction bypass ZipA suggesting a model in which FtsA's self-interaction competes with its ability to recruit downstream division proteins. *Mol. Microbiol.* **83,** 151–167 (2012).

7. Pichoff, S., Du, S. & Lutkenhaus, J. The bypass of ZipA by overexpression of FtsN requires a previously unknown conserved FtsN motif essential for FtsA-FtsN interaction supporting a model in which FtsA monomers recruit late cell division proteins to the Z ring. *Mol. Microbiol.* **95,** 971–987 (2015).

8. Thanedar, S. & Margolin, W. FtsZ Exhibits Rapid Movement and Oscillation Waves in Helix-like Patterns in Escherichia coli. *Curr. Bio* **14,** 1167–1173 (2004).

9. Yang, X. *et al.* GTPase activity–coupled treadmilling of the bacterial tubulin FtsZ organizes

septal cell wall synthesis. *Science* **355,** 744–747 (2017).

10. Jamroškovič, J., Pavlendová, N., Muchová, K., Wilkinson, A. J. & Barák, I. An oscillating Min system in Bacillus subtilis influences asymmetrical septation during sporulation. *Microbiology* **158,** 1972–1981 (2012).

11. Li, G. & Young, K. D. Isolation and identification of new inner membrane-associated proteins that localize to cell poles in Escherichia coli. *Mol. Microbiol.* **84,** 276–295 (2012).

12. Jaffé, A., D'Ari, R. & Hiraga, S. Minicell-forming mutants of Escherichia coli: production of minicells and anucleate rods. *J. Bacteriol.* **170,** 3094–3101 (1988).

13. Mulder, E., El'Bouhali, M., Pas, E. & Woldringh, C. L. The Escherichia coli minB mutation resembles gyrB in defective nucleoid segregation and decreased negative supercoiling of plasmids. *Mol. Genet. Genomics* **221,** 87–93 (1990).

14. Åkerlund, T., Bernander, R. & Nordström, K. Cell division in Escherichia coli minB mutants. *Mol. Microbiol.* **6,** 2073–2083 (1992).

15. Jaffé, A., Vinella, D. & D'Ari, R. The Escherichia coli histone-like protein HU affects DNA initiation, chromosome partitioning via MukB, and cell division via MinCDE. *J. Bacteriol.* **179,** 3494–3499 (1997).

16. Åkerlund, T., Gullbrand, B. & Nordström, K. Effects of the Min system on nucleoid segregation in Escherichia coli. *Microbiology* **148,** 3213–3222 (2002).

17. Jia, S. *et al.* Effect of the Min system on timing of cell division in Escherichia coli. *PLoS One* **9,** e103863 (2014).

18. Loose, M., Fischer-Friedrich, E., Ries, J., Kruse, K. & Schwille, P. Spatial Regulators for Bacterial Cell Division Self-Organize into Surface Waves in Vitro. *Science* **320,** 789–792 (2008).

19. Zieske, K., Schweizer, J. & Schwille, P. Surface topology assisted alignment of Min protein

waves. *FEBS Lett.* **588,** 2545–2549 (2014).

20. Loose, M. & Mitchison, T. J. The bacterial cell division proteins FtsA and FtsZ self-organize into dynamic cytoskeletal patterns. *Nat. Cell Biol.* **16,** 38–46 (2014).

21. Glock, P. *et al.* Optical Control of a Biological Reaction–Diffusion System. *Angew. Chemie Int. Ed.* **57,** 2362–2366 (2018).

22. Osawa, M., Anderson, D. E. & Erickson, H. P. Reconstitution of contractile FtsZ rings in liposomes. *Science* **320,** 792–794 (2008).

23. Shih, Y. L., Fu, X., King, G. F., Le, T. & Rothfield, L. Division site placement in E.coli: Mutations that prevent formation of the MinE ring lead to loss of the normal midcell arrest of growth of polar MinD membrane domains. *EMBO J.* **21,** 3347–3357 (2002).

24. de Boer, P. A., Crossley, R. E., Hand, A. R. & Rothfield, L. I. The MinD protein is a membrane ATPase required for the correct placement of the Escherichia coli division site. *EMBO J.* **10,** 4371–4380 (1991).

25. Devaux, P. F. & Seigneuret, M. Specificity of lipid-protein interactions as determined by spectroscopic techniques. *Biochim. Biophys. Acta - Rev. Biomembr.* **822,** 63–125 (1985).

26. Cornell, R. B. & Taneva, S. G. Amphipathic helices as mediators of the membrane interaction of amphitropic proteins, and as modulators of bilayer physical properties. *Curr. Protein Pept. Sci.* **7,** 539–552 (2006).

27. Zhou, H. & Lutkenhaus, J. Membrane binding by MinD involves insertion of hydrophobic residues within the C-terminal amphipathic helix into the bilayer. *J. Bacteriol.* **185,** 4326–4335 (2003).

28. Hu, Z., Gogol, E. P. & Lutkenhaus, J. Dynamic assembly of MinD on phospholipid vesicles regulated by ATP and MinE. *Proc. Natl. Acad. Sci. U. S. A.* **99,** 6761–6766 (2002).

29. Shih, Y.-L. L. *et al.* The N-Terminal Amphipathic Helix of the Topological Specificity Factor MinE Is Associated with Shaping Membrane Curvature. *PLoS One* **6,** e21425 (2011).

30. Mazor, S. *et al.* Mutual effects of MinD-membrane interaction: I. Changes in the membrane properties induced by MinD binding. *Biochim. Biophys. Acta - Biomembr.* **1778,** 2496–2504 (2008).

31. Vecchiarelli, A. G., Li, M., Mizuuchi, M. & Mizuuchi, K. Differential affinities of MinD and MinE to anionic phospholipid influence Min patterning dynamics in vitro. *Mol. Microbiol.* **93,** 453–463 (2014).

32. Mileykovskaya, E. *et al.* Effects of phospholipid composition on MinD-membrane interactions in vitro and in vivo. *J. Biol. Chem.* **278,** 22193–22198 (2003).

33. Renner, L. D. & Weibel, D. B. MinD and MinE interact with anionic phospholipids and regulate division plane formation in Escherichia coli. *J. Biol. Chem.* **287,** 38835–38844 (2012).

34. Mazor, S. *et al.* Mutual effects of MinD-membrane interaction: II. Domain structure of the membrane enhances MinD binding. *Biochim. Biophys. Acta - Biomembr.* **1778,** 2505–2511 (2008).

35. Mileykovskaya, E. & Dowhan, W. Visualization of Phospholipid Domains in Escherichia coli by Using the Cardiolipin-Specific Fluorescent Dye 10- N -Nonyl Acridine Orange. *J Bacteriol* **182,** 1172–1175 (2000).

36. Fishov, I. & Woldringh, C. L. Visualization of membrane domains in Escherichia coli. *Mol. Microbiol.* **32,** 1166–1172 (1999).

37. Dowhan, W., Mileykovskaya, E. & Bogdanov, M. Diversity and versatility of lipid-protein interactions revealed by molecular genetic approaches. *Biochim. Biophys. Acta - Biomembr.* **1666,** 19–39 (2004).

38. Zieske, K. & Schwille, P. Reconstitution of self-organizing protein gradients as spatial cues in

cell-free systems. *Elife* **3,** e03949 (2014).

39. Schweizer, J. *et al.* Geometry sensing by self-organized protein patterns. *Proc. Natl. Acad. Sci. U. S. A.* **109,** 15283–15288 (2012).

40. Wu, F., van Schie, B. G. C., Keymer, J. E. & Dekker, C. Symmetry and scale orient Min protein patterns in shaped bacterial sculptures. *Nat. Nanotechnol.* **10,** 719–726 (2015).

41. Magde, D., Elson, E. & Webb, W. W. Thermodynamic fluctuations in a reacting system measurement by fluorescence correlation spectroscopy. *Phys. Rev. Lett.* **29,** 705–708 (1972).

42. Rigler, R., Mets, Ü., Widengren, J. & Kask, P. Fluorescence correlation spectroscopy with high count rate and low background: analysis of translational diffusion. *Eur. Biophys. J.* **22,** 169–175 (1993).

43. Eigen, M. & Rigler, R. Sorting single molecules: application to diagnostics and evolutionary biotechnology. *Proc. Natl. Acad. Sci.* **91,** 5740–5747 (1994).

44. Weidemann, T. *et al.* Counting nucleosomes in living cells with a combination of fluorescence correlation spectroscopy and confocal imaging. *J. Mol. Biol.* **334,** 229–240 (2003).

45. Digman, M. A., Dalal, R., Horwitz, A. F. & Gratton, E. Mapping the number of molecules and brightness in the laser scanning microscope. *Biophys. J.* **94,** 2320–2332 (2008).

46. Politi, A. Z. *et al.* Quantitative mapping of fluorescently tagged cellular proteins using FCS-calibrated four-dimensional imaging. *Nat. Protoc.* **13,** 1445–1464 (2018).

47. Petrášek, Z. & Schwille, P. Precise measurement of diffusion coefficients using scanning fluorescence correlation spectroscopy. *Biophys. J.* **94,** 1437–1448 (2008).

48. Einstein, A. Über die von der molekularkinetischen Theorie der Wärme geforderte Bewegung von in ruhenden Flüssigkeiten suspendierten Teilchen. *Ann. Phys.* **322,** 549–560 (1905).

49. von Smoluchowski, M. Zur kinetischen Theorie der Brownschen Molekularbewegung und der

Suspensionen. *Ann. Phys.* **326,** 756–780 (1906).

50. Sutherland, W. LXXV. *A dynamical theory of diffusion for non-electrolytes and the molecular mass of albumin*. *Philos. Mag. Ser. 6* **9,** 781–785 (1905).

51. Thompson, N. L. in *Chemistry and Physics of Lipids* (ed. Lakowicz, J. R.) **50,** 337–378 (Springer US, 1999).

52. Salje, J., van den Ent, F., de Boer, P. & Löwe, J. Direct Membrane Binding by Bacterial Actin MreB. *Mol. Cell* **43,** 478–487 (2011).

53. Pichoff, S. & Lutkenhaus, J. Tethering the Z ring to the membrane through a conserved membrane targeting sequence in FtsA. *Mol. Microbiol.* **55,** 1722–1734 (2005).

54. Parlitz, R. *et al.* Escherichia coli signal recognition particle receptor FtsY contains an essential and autonomous membrane-binding amphipathic helix. *J. Biol. Chem.* **282,** 32176–32184 (2007).

55. Bajar, B. T. *et al.* Improving brightness and photostability of green and red fluorescent proteins for live cell imaging and FRET reporting. *Sci. Rep.* **6,** 20889 (2016).

56. Szeto, T. H., Rowland, S. L., Habrukowich, C. L. & King, G. F. The MinD membrane targeting sequence is a transplantable lipid-binding helix. *J. Biol. Chem.* **278,** 40050–40056 (2003).
