# Supplementary material for: The MinDE system is a generic spatial cue for membrane protein distribution in vitro
Source: Nat Commun. 2018 Sep 26;9:3942. doi: 10.1038/s41467-018-06310-1 (PMC6158289; doi:10.1038/s41467-018-06310-1)
Supplement: Supplementary file 3 — Description of Additional Supplementary Files [file 41467_2018_6310_MOESM3_ESM.pdf]

Description of Additional Supplementary Files

File Name: Supplementary Movie 1
Description: Time-lapse of mCh-MTS(BsD) dynamics in absence and presence of MinDE corresponding to image frames shown in Figure 1a,b. In the absence of MinDE mCh-MTS(BsD) is homogenously distributed (1 µm mCh-MTS(BsD)). In the presence of MinDE and ATP mCh-MTS(BsD) forms traveling surface waves that are anticorrelated to the MinDE wave (1 µM mCh-MTS(BsD), 1 µM MinD (30 % EGFP-MinD), 1 µM MinE). Scale bar: 50 µm

File Name: Supplementary Movie 2
Description: Spatiotemporal regulation of different mCh-MTS constructs by MinDE corresponding to images frames shown in Figure 2b). From left to right MinDE self-organization in the presence of His-mCh, MTS(1xMreB)-mCh, mCh-MTS(FtsA), MTS(FtsY)-mCh, mCh-MTS(BsD), MTS(2xMreB)-mCh. (1 µM MinD (30 % EGFPMinD),1 µM MinE, 1 µM respective mCh-MTS construct). Brightness/contrast of the mCherry channel is not comparable between mCh-MTS constructs. Scale bar: 50 µm

File Name: Supplementary Movie 3
Description: MinDE-dependent spatiotemporal regulation of mCh-MTS constructs containing one or two copies of the *E. coli* MinD MTS (corresponding to image frames shown in Supplementary Fig. 7). From left to right MinDE self-organization in the presence of His-mCh, mCh-MTS(1xMinD), mCh-MTS(2xMinD) and the dimerizing mCh-Jun-MTS(2xMinD) (1 µM MinD (30 % EGFP-MinD), 1 µM MinE, 1 µM mCh-MTS). Brightness/contrast of the mCherry channel is not comparable between mCh-MTS constructs. Scale bar: 50 µm

File Name: Supplementary Movie 4
Description: MinDE self-organization spatiotemporally regulates streptavidin bound to biotinylated lipids on a SLB (corresponding to image frames shown in Figure 3a) (1 µM MinD, 1 µM MinE, Alexa647-streptavidin). Scale bar: 50 µm

File Name: Supplementary Movie 5
Description: Large scale streptavidin gradient formation by MinDE is reversible (corresponding to image frames shown in Figure 3e). Addition of 2.5 mM Na_3VO_4 to an established MinDE self-organization assay (1 µM MinD, 1 µM MinE, alexa647-streptavidin) leads to MinDE detachment (left panel) and subsequent homogenization of streptavidin fluorescence (right panel) on the membrane. Addition of Na_3VO_4 at t= 161 s. Scale Bar: 50 µm

File Name: Supplementary Movie 6
Description: Time-lapse of MinDE oscillations and streptavidin counteroscillations in PDMS microcompartments (corresponding to image frames shown in 4b) (1 µM MinD (30 % EGFP-MinD), 2 µM MinE, Alexa647-streptavidin). Brightness of the streptavidin channel was corrected for bleaching using histogram matching in Fiji. Scale bar: 10 µm

File Name: Supplementary Movie 7

Description: Time-lapse of MinDE oscillations and mCh-MTS(BsD) counteroscillations in PDMS microcompartments (corresponding to image frames shown in Figure 4e) (1 µM MinD (30 % EGFP-MinD), 2 µM MinE, 0.5 µM mCh-MTS(BsD)). Scale bar: 10 µm

File Name: Supplementary Movie 8
Description: Time-laps of MinDE self-organization in the presence of FtsA (corresponding to image frames shown in Supplementary Fig. 15) (0.4 µM Cy5-FtsA, 1 µM MinD (30 % EGFP-MinD), 1 µM MinE). ATP is added at t=0 s to start self-organization. Scale bar: 50 µm

File Name: Supplementary Movie 9
Description: MinC enhances spatiotemporal regulation of FtsZ-YFP-MTS by MinDE. Time-lapse of MinDE self-organization in the presence of FtsZ-YFP-MTS (corresponding to images shown in Fig. 5ab). Top left: High free Mg^{2+} without MinC. Top right: High free Mg^{2+} with MinC. Bottom left: Low free Mg^{2+} without MinC. Bottom right: Low free Mg^{2+} with MinC (1 µM MinD (30 % EGFP-MinD), 1 µM MinE, 0.5 µM FtsZ-YFP-MTS, with and without 0.05 µM MinC). All images of the same spectral channel are acquired and displayed using the same imaging and brightness and contrast settings.

File Name: Supplementary Movie 10
Description: MinDE regulate FtsZ-YFP-MTS attachment, but not its lateral diffusion. Time-lapse of MinDE self-organization in the presence of FtsZ-YFP-MTS (corresponding to images shown in Supplementary Fig. 16). Top left: High free Mg^{2+} without MinC. Top right: High free Mg^{2+} with MinC. Bottom left: Low free Mg^{2+} without MinC. Bottom right: Low free Mg^{2+} with MinC (1 µM MinD (30 % EGFP-MinD), 1 µM MinE, 0.5 µM FtsZ-YFP-MTS, with and without 0.05 µM MinC). Instrumental settings are not comparable between images.

File Name: Supplementary Movie 11
Description: Time-series of MinDE self-organization spatiotemporally regulating a 30 bp dsDNA labeled with Alexa647 and bound to the membrane by a cholesterol anchor corresponding to image frames shown in Figure 6a) (1 µM MinD (30% EGFP-MinD), 1 µM MinE, 10nM TEG-choleterol-dsP1). Scale bar: 50 µm

File Name: Supplementary Movie 12
Description: Time-series of MinDE self-organization spatiotemporally regulating a 300 bp dsDNA bound to lipid-anchored streptavidin corresponding to image frames shown in Figure 6b) (1 µM MinD (30 % EGFPMinD), 2 µM MinE, streptavidin, 300 bp lambda DNA). Scale bar: 50 µm

File Name: Supplementary Movie 13
Description: Time-series of MinDE self-organization spatiotemporally regulating a 2000 bp dsDNA bound to lipid-anchored streptavidin corresponding to image frames shown in Figure 6c) (1 µM MinD (30 % EGFPMinD), 2 µM MinE, streptavidin, 2000 bp lambda DNA). Scale bar: 50 µm

File Name: Supplementary Movie 14
Description: Direct comparison between spatiotemporal regulation of mCh-MTS(BsD) and lipidanchored streptavidin by MinDE. Left: Time-series of colliding MinDE waves in the

presence of mCh-MTS(BsD) (corresponding to image frames shown in Figure 7a). (1 µM MinD (30 % EGFP-MinD), 1 µM MinE, 1 µM mCh-MTS(BsD)). Right: Time-series of colliding MinDE waves in the presence of streptavidin bound to biotinylated lipids corresponding to image frames shown in Figure 7b) (1 µM MinD (30 % EGFP-MinD), 1 µM MinE, Alexa647-streptavidin). Streptavidin cannot dissociate in solution and is moved laterally on the membrane leading to accumulation on collision interfaces and depletion in spiral centers. Scale bars: 50 µm
